# Supplementary material for: DG-LLM: Decomposition-based dynamic graph adaptation of large language models for spatiotemporal traffic forecasting
Source: PLoS One. 2026 May 19;21(5):e0349527. doi: 10.1371/journal.pone.0349527 (PMC13186391; doi:10.1371/journal.pone.0349527)
Supplement: S1 Appendix — PDF file containing the mathematical formulations and background for Graph Neural Networks (GNNs), Variational Mode Decomposition (VMD), and the Transformer architecture used in this study. (PDF) [file pone.0349527.s001.pdf]

S1 Appendix: Preliminaries

Graph Neural Networks

Graph Neural Networks (GNNs) are a class of deep learning architectures that extend classical deep learning to graph data. They allow information to be propagated between nodes on a graph. One such formulation is the graph convolution on a spatial graph:

$$H^{(\ell+1)} = \phi\Big(\tilde{A}H^{(\ell)}W^{(\ell)}\Big), \tag{1}$$

where $H^{(\ell)} \in \mathbb{R}^{N\times d_\ell}$ are node features on a graph G at layer ℓ, $\tilde{A}$ is a normalized adjacency matrix (for example, $D^{-\frac{1}{2}}AD^{-\frac{1}{2}}$), $W^{(\ell)}$ is a weight matrix, and $\phi(\cdot)$ is a non-linear activation function. Although effective in static graphs, it assumes a fixed spatial relationship and cannot handle time-dependent relationships.

Graph Attention Networks (GATs) address this limitation by learning adaptive, data-driven edge weights that reflect the relative importance of neighboring nodes [1]. Given node features h_i and h_j, a shared linear transformation is first applied, followed by an attention mechanism:

$$e_{ij} = a^\top \mathrm{LeakyReLU}(W[h_i \,\|\, h_j])\,, \tag{2}$$

where W and a are learnable parameters. The attention scores are normalized across the neighborhood of the node i using a softmax:

$$\alpha_{ij} = \frac{\exp(e_{ij})}{\sum_{k\in\mathcal{N}(i)}\exp(e_{ik})}. \tag{3}$$

The updated node representation is obtained by aggregating neighboring features weighted by the learned attention coefficients:

$$h_i' = \sigma\left(\sum_{j\in\mathcal{N}(i)} \alpha_{ij}Wh_j\right), \tag{4}$$

where $\sigma(\cdot)$ is a non-linear activation function. Multi-head attention can be applied to stabilize training and capture diverse interaction patterns, with the outputs either concatenated or averaged as in the original GAT formulation.

Variational Mode Decomposition (VMD)

The time series of traffic volumes has multi-scale temporal characteristics with long-term trends, periodic behaviors (e.g., daily or weekly periodicity), and irregular fluctuations over short periods of time. To separate these time series with multiple time scales, we use a method called Variational Mode Decomposition (VMD) [2]. VMD decomposes an input time series $f(t)$ into a set of discrete modes called Intrinsic Mode Functions (IMFs):

$$f(t) = \sum_{k=1}^{K} u_k(t), \tag{5}$$

where each mode $u_k(t)$ is assumed to be concentrated around a specific center frequency ω_k. VMD formulates the decomposition as a constrained optimization problem. The objective is to minimize the sum of the estimated bandwidths of each mode, subject to the constraint that the modes perfectly reconstruct the original signal:

$$\min_{u_k,,\omega_k} \sum_{k=1}^{K} \left|\partial_t \left[\left(\delta(t) + \frac{j}{\pi t}\right) * u_k(t)\right] e^{-j\omega_k t}\right| 2^2, \quad \text{s.t.} \ \sum k = 1^K u_k(t) = f(t). \tag{6}$$

Here, the term $\left(\delta(t) + \frac{j}{\pi t}\right) * u_k(t)$ represents the analytic signal after the Hilbert transform, and the exponential term $e^{-j\omega_k t}$ is a heterodyne operation that shifts the spectrum of the mode frequency to baseband. To solve this problem, the unaugmented Lagrangian formulation is employed with a quadratic penalty

factor α and Lagrange multipliers λ. Here, α is a bandwidth constraint that balances the reconstruction quality with the spectral smoothness of the modes. The solution is iteratively computed by the Alternate Direction Method of Multipliers (ADMM). The iterations are performed until the relative difference between the updated modes and the previous modes is less than a specified convergence tolerance ϵ:

$$\sum_{k=1}^{K} \frac{|u_k^{n+1} - u_k^n|_2^2}{|u_k^n|_2^2} < \epsilon. \tag{7}$$

For a traffic network with N nodes, VMD is applied independently to the temporal signal at each node. This yields a mode-specific tensor representation given by:

$$\mathbf{U}k(t) = \left[u1, k(t), u_{2,k}(t), \ldots, u_{N,k}(t)\right]^\top \in \mathbb{R}^N, \tag{8}$$

where $k = 1, \ldots, K$. Each mode in the representation captures traffic patterns at a different temporal scale, from low-frequency global patterns to high-frequency local noise. This is a more stable and well-separated feature representation for use in downstream forecasting models.

Transformer Architecture and Self-Attention

Transformers are sequence modeling techniques that use self-attention mechanisms. Self-attention allows any position in the sequence to attend to all the other positions by solving the gradient vanishing problem. Therefore, Transformers are efficient in handling long-range dependencies. Self-attention mechanisms are important in time-series and traffic flow forecasting, as the future state of the system might depend on recent and distant past contexts.

The basic attention mechanism is called the Scaled Dot-Product Attention. Let Q, K, and V be the matrices of the query, key, and value vectors, respectively. The attention is given by

$$\text{Attention}(Q, K, V) = \text{softmax}\left(\frac{QK^\top}{\sqrt{d_k}}\right) V, \tag{9}$$

where d_k is the key dimension. The scaling factor is used to stabilize training by controlling large dot product values.

To account for different patterns of interactions, a Multi-Head Attention is used by the Transformers. The input is mapped into different representation subspaces, and then attention is applied:

$$\text{MultiHead}(H) = \text{Concat}(\text{head}_1, \ldots, \text{head}_h) W^O, \tag{10}$$

where each attention head_i is computed using Eq. (9). This mechanism enables the model to attend to information from different positions and feature subspace simultaneously.

Each Transformer layer further includes a position-wise feed-forward network (FFN) with residual connections and layer normalization:

$$\text{FFN}(x) = \max(0, xW_1 + b_1)W_2 + b_2, \tag{11}$$

$$H' = \text{LN}(H + \text{MultiHead}(H)), \quad H'' = \text{LN}(H' + \text{FFN}(H')). \tag{12}$$

Pretrained large language models (LLMs) are built upon this Transformer architecture and benefit from large-scale pretraining that yields strong sequence modeling and relational representation learning.

Partially Frozen Attention

While the complete fine-tuning of the Transformer model is computationally expensive and can result in the loss of pre-trained representations due to catastrophic forgetting, existing studies have shown that the lower-level representations contain some generalized temporal features. To leverage this property, we adopt a partially frozen attention strategy to update only a subset of attention parameters during task-specific adaptation, motivated by the STLLM framework [3].

Let $L = F+U$ denote the total number of Transformer layers, where F is the total number of frozen layers, and U is the total number of unfrozen layers. The parameters are frozen for layers $\ell \leq U$. The parameters are unfrozen only for the attention projection matrices (W^Q, W^K, W^V, W^O) for layers $\ell > U$, while the feed-forward networks are frozen. This configuration maintains the pre-trained temporal representations while enabling the upper layers to adjust for task-dependent dependencies with minimal computational cost.

Low-Rank Adaptation (LoRA)

Pre-trained neural networks typically use dense, full-rank weight matrices. Nonetheless, past studies have indicated that large language models can adapt within a low-dimensional subspace, suggesting a low intrinsic dimensionality. Low Rank Adaptation, abbreviated as LoRA, limits the weight update during the fine-tuning of neural networks to a low-rank form [4].

Given a pretrained weight matrix $W_0 \in \mathbb{R}^{d\times k}$, the update is parameterized as:

$$W = W_0 + \Delta W = W_0 + BA, \tag{13}$$

where $B \in \mathbb{R}^{d\times r}$, $A \in \mathbb{R}^{r\times k}$, and $r \ll \min(d, k)$. During training, W_0 is frozen, and only A and B are updated.

For a given input vector x, the forward pass is given by:

$$h = W_0 x + \Delta W x = W_0 x + BAx. \tag{14}$$

The low-rank matrices are initialized such that $\Delta W = 0$ to start with, with A being sampled from a Gaussian distribution and B being set to zero. The update is further scaled by a factor α/r, where α is a constant. This scaling stabilizes optimization and reduces sensitivity to the choice of rank r.

References

[1] Veličković P, Cucurull G, Casanova A, Romero A, Lio P, Bengio Y. Graph attention networks. arXiv preprint arXiv:171010903. 2017.

[2] Dragomiretskiy K, Zosso D. Variational mode decomposition. IEEE Transactions on Signal Processing. 2013;62(3):531-44.

[3] Liu C, Yang S, Xu Q, Li Z, Long C, Li Z, et al. Spatial-temporal large language model for traffic prediction. In: 2024 25th IEEE International Conference on Mobile Data Management (MDM). IEEE; 2024. p. 31-40.

[4] Hu EJ, Shen Y, Wallis P, Allen-Zhu Z, Li Y, Wang S, et al. Lora: Low-rank adaptation of large language models. ICLR. 2022;1(2):3.
