# Supplementary material for: DG-LLM: Decomposition-based dynamic graph adaptation of large language models for spatiotemporal traffic forecasting
Source: PLoS One. 2026 May 19;21(5):e0349527. doi: 10.1371/journal.pone.0349527 (PMC13186391; doi:10.1371/journal.pone.0349527)
Supplement: Attachment — Submitted filename: Review Plosone.pdf [file pone.0349527.s002.pdf]

The manuscript addresses an important problem in spatiotemporal traffic forecasting and proposes an interesting combination of signal decomposition, dynamic graph learning, and large language models. The experimental evaluation is extensive and conducted on multiple real-world datasets, which is a strong aspect of the work.

However, several major issues need to be addressed before the paper can be considered for publication.

First, the scientific novelty is not clearly articulated. The proposed framework appears to be mainly a combination of existing techniques, and the authors should explicitly clarify what is fundamentally new in their approach.

Second, the justification for using a pretrained LLM (GPT-2) is weak. It is not convincingly demonstrated why an LLM is more suitable than standard Transformer-based time series models. A clearer motivation and, if possible, additional comparative analysis are needed.

Third, the statistical analysis lacks rigor. While MAE, RMSE, and MAPE are reported, no statistical significance tests or confidence intervals are provided, making it difficult to assess whether the reported improvements are meaningful.

Fourth, the model architecture is very complex, which raises concerns about overfitting and practical applicability. The authors should discuss the trade-off between model complexity and performance gains.

Overall, the paper has potential, but substantial revision is required to clarify the contribution, strengthen the methodology, and improve the rigor of the experimental analysis.
