# Supplementary material for: DG-LLM: Decomposition-based dynamic graph adaptation of large language models for spatiotemporal traffic forecasting
Source: PLoS One. 2026 May 19;21(5):e0349527. doi: 10.1371/journal.pone.0349527 (PMC13186391; doi:10.1371/journal.pone.0349527)
Supplement: Attachment — Submitted filename: Response to reviewers.pdf [file pone.0349527.s003.pdf]

Response to Reviewers' Comments

Journal:	PLOS ONE
Manuscript ID:	PONE-D-26-00024
Title of Paper:	DG-LLM: Decomposition-based dynamic graph adaptation of large language models for spatiotemporal traffic forecasting
Authors:	Sadia Tabassum, Naushin Nower
Date Sent:	February 6, 2026

Thank you immensely for your thorough and constructive review of our manuscript. Your insightful observations have significantly contributed to refining the quality and clarity. In response to your feedback, we have diligently addressed each point, making precise modifications throughout the revised manuscript. To ensure easy identification, all revisions have been highlighted in blue within the revised manuscript. We deeply appreciate the opportunity to refine our work and are committed to addressing the reviewers' concerns effectively.

Reviewer #1:

The manuscript addresses an important problem in spatiotemporal traffic forecasting and proposes an interesting combination of signal decomposition, dynamic graph learning, and large language models. The experimental evaluation is extensive and conducted on multiple real-world datasets, which is a strong aspect of the work. However, several major issues need to be addressed before the paper can be considered for publication.

First, the scientific novelty is not clearly articulated. The proposed framework appears to be mainly a combination of existing techniques, and the authors should explicitly clarify what is fundamentally new in their approach.

Response:

We thank the reviewer for their valuable feedback. We agree that the original manuscript did not sufficiently emphasize the core scientific novelty. While our framework builds upon existing components, the novelty lies in their joint modelling to address the overlooked problem of frequency-dependent spatial heterogeneity in spatiotemporal forecasting. Specifically, we observe that spatial dependencies vary across temporal modes (e.g., trend, periodicity, and high-frequency components), a factor that is not explicitly modeled in existing approaches.

Contrary to existing models that either directly apply LLMs to the entangled time series or use static graphs, the proposed DG-LLM introduces a mode-dependent dynamic graph learning mechanism. In particular, we use Variational Mode Decomposition (VMD) to explicitly extract the intrinsic modes of the time series (i.e., trend, daily periodicity, and high-frequency noise), and show that each mode exhibits distinct spatial dependency patterns. Based on this observation, we design a dynamic graph learning module that learns separate, mode-specific graph topologies, which are then integrated into the LLM for spatiotemporal modeling.

We have revised the Introduction section (Page 3) of the manuscript to explicitly highlight the proposed modeling paradigm. The added paragraph is provided below:

DG-LLM proposes a new paradigm for spatiotemporal forecasting by simultaneously disentangling frequency-specific temporal patterns and mode-specific spatial structures before employing the LLM for spatiotemporal prediction. Unlike previous approaches that employed LLMs on raw, entangled time series data or adopted a static graph, we leverage Variational Mode Decomposition (VMD) as a task-aware pre-processing component to disentangle the time series into intrinsic modes before the LLM models temporal patterns. As a result, the proposed approach can explicitly extract the intrinsic modes of the time series (i.e., trend, daily periodicity, and high-frequency noise). We further propose a mode-dependent dynamic spatial routing mechanism that learns separate graph topologies for each time series mode, as spatial correlations are not homogeneous across frequencies; long-range trends and high-frequency noise have different dependency structures. By jointly modeling decomposed temporal patterns and their corresponding spatial structures, we capture multi-scale dependencies more effectively than existing approaches that rely on raw signals or static graph representations,

thereby improving generalization across diverse datasets.

Second, the justification for using a pretrained LLM (GPT-2) is weak. It is not convincingly demonstrated why an LLM is more suitable than standard Transformer-based time series models. A clearer motivation and, if possible, additional comparative analysis are needed.

Response:

We would like to express our gratitude to the reviewer for their constructive critique. The main reason for using a pretrained LLM like GPT-2 instead of a standard Transformer model is its ability to learn rich and transferable sequence representations from large-scale pretraining. In our framework, the model operates on decomposed temporal modes combined with dynamically learned spatial structures, which introduces complex and heterogeneous patterns. Standard Transformer-based time series models, typically trained from scratch, may struggle to generalize under such conditions, especially in the presence of limited data and distribution shifts.

To validate this, we conducted a comparative analysis across multiple Transformer-based backbones, including both pretrained and non-pretrained variants. Specifically, we evaluated GPT-2 (pretrained and non-pretrained), BERT, DistilGPT-2, and T5. The result table is shown below:

***Table**: Comparative Analysis Across Different Pretrained and Non-Pretrained Backbones*

Backbone	MAE	MAPE	RMSE
T5 (not pretrained)	6.7799	47.17%	12.6522
GPT2 (not pretrained)	5.3352	38.61%	9.4408
BERT	5.3949	36.47%	9.8098
**GPT2 (our choice)**	**5.0742**	**36.17%**	**9.2021**
DistilGPT2	5.202	36.34%	9.5349

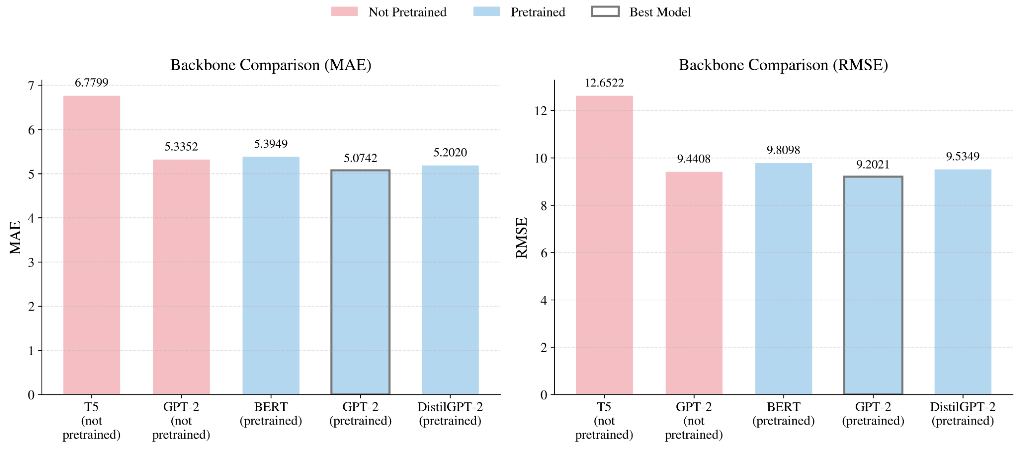

***Figure**: MAE and RMSE Comparison on Different Pretrained and Non-Pretrained Backbones*

The results are presented in Figure 11 (Page 22) of the revised manuscript. It shows that the

pre-trained GPT-2 significantly outperforms its non-pretrained counterpart as well as other Transformer variants. This indicates that the improvements are not due to the Transformer architecture itself, but rather to the latent universal knowledge acquired during the LLM's pre-training. We have added a subsection, Sensitivity to Backbone Choice (Page 21), in the discussion section as below-

Sensitivity to Backbone Choice: Beyond numerical hyperparameters, the choice of the pre-trained backbone is critical for capturing temporal dependencies. We compare the performance of the pre-trained GPT-2 model, the pre-trained BERT model, the pre-trained DistilGPT-2 model, the GPT-2 model without pre-training, and the T5 encoder-decoder model without pre-training. As depicted in Fig. 11, the performance of the models with pre-training is comparatively better than that of the models without pre-training. Among all the models, the pre-trained GPT-2 achieves the best performance on the NYCTaxi dataset, with an MAE of 5.07 and an RMSE of 9.20. On the other hand, models trained without pretraining, including the randomly initialized GPT-2, exhibit substantially higher errors, indicating that architectural similarity alone is insufficient without pretrained knowledge. Although other pre-trained models, such as BERT and DistilGPT-2, achieve competitive results, the pre-trained GPT-2 model still performs slightly better. This is because BERT is an encoder-only model designed for bidirectional context, whereas the DistilGPT-2 model's performance is limited by its capacity. This makes the pre-trained GPT-2 model an optimal choice for learning the temporal dependencies.

Third, the statistical analysis lacks rigor. While MAE, RMSE, and MAPE are reported, no statistical significance tests or confidence intervals are provided, making it difficult to assess whether the reported improvements are meaningful.

Response:

We thank the reviewer for highlighting the importance of rigorous statistical evaluation. We agree that the original manuscript lacked sufficient statistical analysis, and we have significantly strengthened this aspect in the revised version.

To address this issue, we have re-run the experiments for all the datasets using five different random seed values. Based on these runs, we now report the mean and 95% confidence intervals for MAE, RMSE, and MAPE as shown below:

Table: *Multiseed Results with 95% interval for all Datasets*

Dataset	MAE Mean	MAE 95% CI	RMSE Mean	RMSE 95% CI	MAPE(%) Mean	MAPE(%) 95% CI
Taxi Drop	5.0594	[5.006, 5.113]	8.5906	[8.498, 8.684]	35.7	[35.28, 36.11]
Taxi Pick	5.2234	[5.180, 5.267]	8.7743	[8.693, 8.856]	34.76	[33.51, 36.00]
Bike Drop	1.6403	[1.630, 1.651]	2.3993	[2.373, 2.425]	45.49	[44.71, 46.26]
Bike Pick	1.7393	[1.726, 1.753]	2.6491	[2.615, 2.683]	48.17	[47.17, 49.17]
PEMSD04	18.8093	[18.19, 19.43]	28.2706	[27.78, 28.76]	13.84	[11.63, 16.05]

PEMSD08	14.5071	[14.20, 14.82]	22.6447	[22.19, 23.10]	10.96	[9.31, 12.60]

In addition, to assess whether the observed improvements are statistically significant, we conducted paired t-tests on the MAE and RMSE values obtained from DG-LLM and the baseline models across all six datasets. The results show that the improvements of DG-LLM are statistically significant ($p < 0.001$) across most datasets. The statistical accuracy values are provided in Figure 5 (Page 19) as given below:

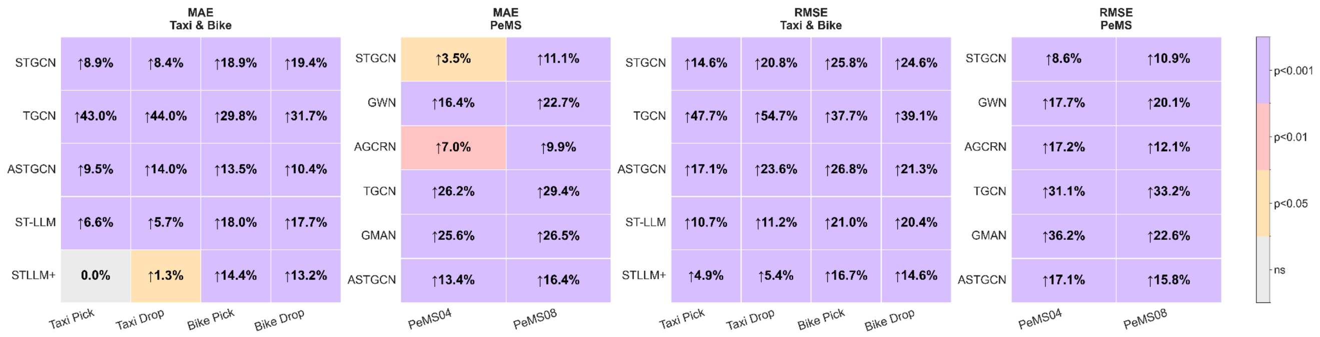

Figure: *Statistical significance of DG-LLM improvements.Heatmaps show percentage reduction in MAE and RMSE relative to baseline models (n=5 seeds), along with corresponding significance levels. Colors denote significance: purple ($p < 0.001$), red ($p < 0.01$), orange ($p < 0.05$), and grey (not significant). Upward arrows (↑) indicate error reduction by DG-LLM.*

We addressed this in our revised manuscript in the Results section (Pages 18-19) as below-

To verify the reliability of the obtained performance improvements, we conduct statistical significance testing for both the average-horizon MAE and RMSE across all datasets, as depicted in Fig. 5. The figure shows the relative improvement of DG-LLM over each baseline together with the corresponding significance level. We observe that all RMSE improvements are statistically significant at $p<0.001$, while MAE improvements are statistically significant for the majority of comparisons. These results indicate that the proposed DG-LLM framework consistently reduces both average prediction error and larger error deviations relative to competing methods. Overall, these results verify that the obtained performance improvements are not due to random variations but demonstrate the robustness and effectiveness of our proposed framework.

Fourth, the model architecture is very complex, which raises concerns about overfitting and practical applicability. The authors should discuss the trade-off between model complexity and performance gains.

Response:

We thank the reviewer for this important comment. While DG-LLM introduces additional components (e.g., VMD decomposition and dynamic graph learning), these modules are designed to better capture the complex spatio-temporal patterns in traffic data. The VMD layer helps separate stable temporal patterns from high-frequency noise, allowing the model to learn from more structured and less

entangled signals. This reduces the burden on the model and mitigates overfitting by separating stable patterns from high-frequency noise.
In addition, the DG-LLM model utilizes a parameter-efficient fine-tuning method (LoRA), in which the majority of the layers of the pre-trained LLM are kept frozen during the fine-tuning process. As shown in Table 9, only 7% of the parameters of the pre-trained model are fine-tunable, while more than 92% of the parameters are frozen, which limits the capability of the pre-trained model during the fine-tuning process. In terms of the trade-off between complexity and performance, we observe that each component of DG-LLM contributes to measurable performance gains, as demonstrated in our ablation studies (Page 20). At the same time, the parameter-efficient design ensures that the additional architectural complexity is moderate. As reported in the revised manuscript, DG-LLM maintains moderate computational complexity (in terms of GFLOPs) and achieves substantial memory savings (approximately 32.7% reduction in RAM usage compared to full fine-tuning). We have added a new subsection, "Computational Efficiency Analysis," on page 22, as given below.

DG-LLM adopts a parameter-efficient fine-tuning strategy, LoRA, in which only a small fraction of the model parameters are updated during training. Although the full model contains approximately 257M parameters, only about 7.15% (~18.4M) are trainable, while the majority of the pre-trained backbone remains frozen as shown in Table 8. This significantly reduces the effective optimization cost compared to full fine-tuning. In addition, Table 8 reports the computational efficiency across datasets, including GFLOPs, training time per epoch, and inference latency. Despite incorporating additional components such as VMD and dynamic graph learning, DG-LLM reduces RAM usage by 32.7% compared to full fine-tuning while maintaining moderate computational complexity and practical runtime performance. These results demonstrate that the proposed framework achieves a favorable balance between model capacity and efficiency for large-scale spatiotemporal forecasting.

***Table**: Computational efficiency of DG-LLM across datasets. Parameter counts are in millions (M).*

Dataset	Nodes	Total Params (M)	Trainable (M)	Train %	GFLOPs	Time/ Epoch (s)	Latency (ms)
Taxi Drop	266	257.78	18.44	7.15%	79.89	176	253
Taxi Pick	266	257.78	18.44	7.15%	79.89	181	260
Bike Drop	250	257.76	18.43	7.15%	74.67	159.6	237
Bike Pick	250	257.76	18.43	7.15%	74.67	165	238
PEMSD04	307	257.81	18.47	7.17%	93.48	829.8	292
PEMSD08	170	257.7	18.37	7.13%	49.4	460	183

Overall, the paper has potential, but substantial revision is required to clarify the contribution, strengthen the methodology, and improve the rigor of the experimental analysis.

Reviewer #2:

This manuscript presents a technically ambitious framework that combines signal decomposition, dynamic graph learning, and LLM adaptation for spatiotemporal traffic forecasting. The idea of injecting learned, mode-specific graph structures into an LLM via constrained attention is interesting, and the experimental scope is broader than many comparable studies.

The main concern is not correctness, but clarity of contribution and empirical rigor. The model is complex, with many interacting components (VMD, dynamic graphs, graph masking, LoRA, residual fusion). While the ablation study shows that removing components degrades performance, it does not clearly explain why each component is necessary or how much benefit it provides relative to its added complexity.

Response:

We would like to express our sincere gratitude to the reviewer for their positive feedback and insightful criticism. We agree that it is important not only to show performance degradation in ablation studies, but also to clearly explain the role of each component and justify the added architectural complexity.

To address this, we have extended the ablation analysis in the revised manuscript by explicitly quantifying and interpreting the contribution of each component. As shown in Figure 7 (Page 20), different modules have varying levels of impact on performance. In particular, removing the dynamic graph learning module results in the largest degradation (up to ~10% on NYCTaxi and ~20% on CHBike), highlighting its critical role in modeling mode-dependent spatial relationships. The spatiotemporal encoding module also shows a substantial impact, while VMD contributes by reducing temporal entanglement and improving learning stability. The LoRA module has comparatively smaller performance gain, but it improves efficiency with minimal added complexity.

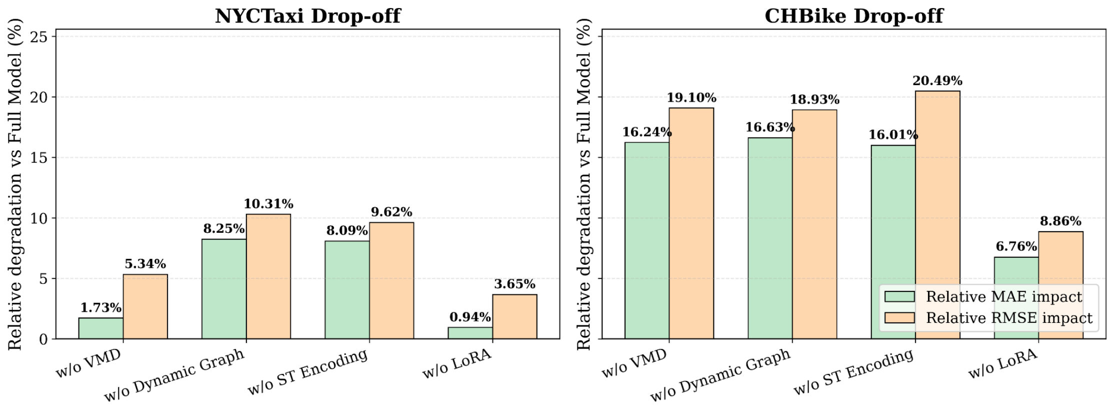

***Figure**: Relative MAE and RMSE impact of ablation studies on the full model*

These results demonstrate that each component addresses a specific limitation (e.g., temporal entanglement, static spatial assumptions, or inefficient fine-tuning) and that the performance gains are concentrated in key modules rather than uniformly distributed.

We have incorporated this detailed analysis into the ablation study discussion in the Results section (Page 20) in detail in the revised manuscript to explicitly clarify the necessity and contribution of each component. We have also expanded the Introduction section (Page 3) to clarify the contribution as follows:

DG-LLM proposes a new paradigm for spatiotemporal forecasting by simultaneously disentangling frequency-specific temporal patterns and mode-specific spatial structures before employing the LLM for spatiotemporal prediction. Unlike previous approaches that employed LLMs on raw, entangled time series data or adopted a static graph, we leverage Variational Mode Decomposition (VMD) as a task-aware pre-processing component to disentangle the time series into intrinsic modes before the LLM models temporal patterns. As a result, the proposed approach can explicitly extract the intrinsic modes of the time series (i.e., trend, daily periodicity, and high-frequency noise). We further propose a mode-dependent dynamic spatial routing mechanism that learns separate graph topologies for each time series mode, as spatial correlations are not homogeneous across frequencies; long-range trends and high-frequency noise have different dependency structures. By jointly modeling decomposed temporal patterns and their corresponding spatial structures, we capture multi-scale dependencies more effectively than existing approaches that rely on raw signals or static/global graph representations, thereby improving generalization across diverse datasets.

The statistical evaluation is another weakness. Improvements are reported without any assessment of variance or significance, which is problematic given the relatively small margins in some comparisons. Running multiple seeds and reporting confidence intervals would substantially strengthen the results.

Response:

We thank the reviewer for highlighting the importance of statistical rigor. We agree that reporting single-run results is insufficient, particularly when performance differences are relatively small. To address these issues, we have comprehensively revised our experimental protocol.

In the revised manuscript, we re-ran all primary experiments using five different random seeds. We report the mean±standard deviation for all the results (MAE, RMSE, MAPE) in the tables (Table 3-5). The results are shown below:

Table*: Multiseed Results with 95% interval for all Datasets*

Dataset	MAE Mean	MAE 95% CI	RMSE Mean	RMSE 95% CI	MAPE(%) Mean	MAPE(%) 95% CI
Taxi Drop	5.0594	[5.006, 5.113]	8.5906	[8.498, 8.684]	35.7	[35.28, 36.11]
Taxi Pick	5.2234	[5.180, 5.267]	8.7743	[8.693, 8.856]	34.76	[33.51, 36.00]

Bike Drop	1.6403	[1.630, 1.651]	2.3993	[2.373, 2.425]	45.49	[44.71, 46.26]
Bike Pick	1.7393	[1.726, 1.753]	2.6491	[2.615, 2.683]	48.17	[47.17, 49.17]
PEMSD04	18.8093	[18.19, 19.43]	28.2706	[27.78, 28.76]	13.84	[11.63, 16.05]
PEMSD08	14.5071	[14.20, 14.82]	22.6447	[22.19, 23.10]	10.96	[9.31, 12.60]

To further support this, we also added a visualization of DG-LLM's performance with 95% confidence intervals across all prediction horizons, as shown in Figure 6 (Page 19). The results demonstrate that DG-LLM consistently achieves lower MAE values than baseline models across all horizons, while maintaining relatively narrow confidence intervals. This indicates that the observed improvements are stable and not due to random fluctuations.

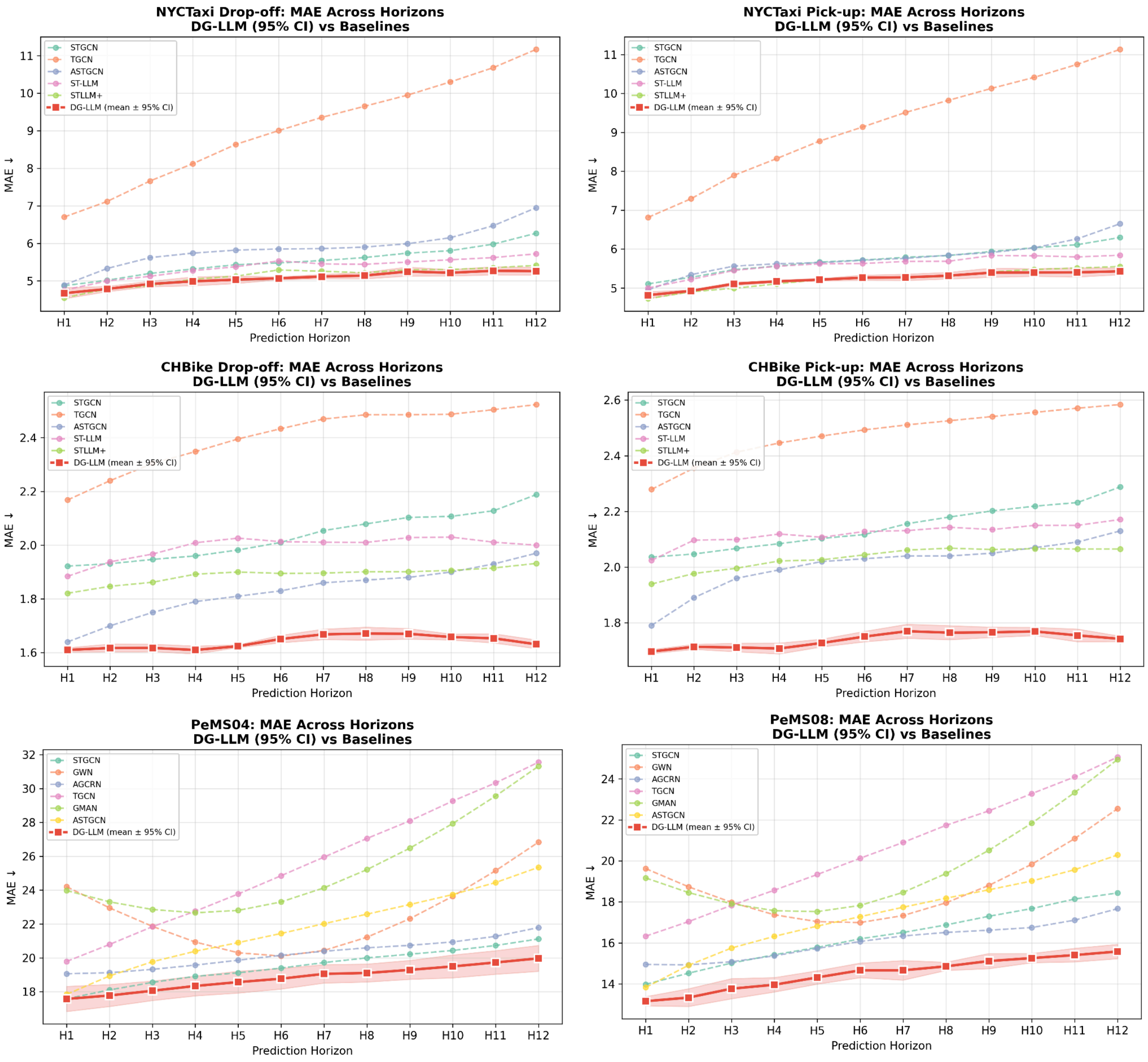

***Figure**: Mean Absolute Error (MAE) comparison of DG-LLM against baseline models across 12 prediction horizons for all datasets. Shaded areas represent the 95% confidence interval.*

We have incorporated these statistical analyses and visualizations into the Results section (Page 19) of the revised manuscript.

Reproducibility is reasonably addressed through the availability of public data and code, but clearer documentation of preprocessing and training configurations would help.

Response:

We thank the reviewer for recognizing our efforts in reproducibility. We agree that clearer documentation of preprocessing and training configurations is important to facilitate reproducibility.

To address this, we have improved both our code repository and manuscript. Specifically, we have revised the README.md and added a dedicated Configuration.md file in our public repository, where we provide detailed descriptions of the data preprocessing pipeline, training procedures, and all hyperparameter settings used in our experiments. This ensures that the results can be reliably reproduced. The link to our code repository is: https://github.com/SadiaTabassum1216/DG-LLM.

Besides, we have an Implementation Details section in the manuscript (Page 15) for the brief configuration description as given below-

All datasets, including NYCTaxi, CHBike, and PeMS, are divided chronologically into training, validation, and test sets with a standard division of 6:2:2. The input sequence length and prediction horizon are set at T_{in} = T_{out} = 12 and 96 time steps, respectively, to test the model's performance in short- and long-term forecasting. Temporal encoding is based on periodicity in each dataset. For the NYCTaxi and CHBike datasets, the periodicity in day T_d is set to 48 time steps, with a sampling time of 30 minutes. For the PeMS dataset, the periodicity in a day, $T_{d,}$ is set at 288 time steps with a sampling time of 5 minutes. For all datasets, the weekly periodicity, T_w, is set to 7.

To improve computational efficiency, VMD is performed with K=3 modes, a bandwidth constraint of α=2000, and a convergence tolerance of 1×10^{-7}. VMD is applied to each window separately using only the input sequence X of length T_{in}, without including any future prediction window Y of length T_{out} to avoid any future information leakage. The core architecture utilizes a six-layer GPT-2 backbone, fine-tuned via LoRA with a rank (r) of 16 and an α of 32. To maintain spatial efficiency in the dynamic graph learning, we apply a sparsity threshold τ, which retains the top 15% learned edges per node. Activation checkpointing is further employed to reduce GPU memory consumption by recomputing intermediate activations during backpropagation.

Finally, some language in the paper overstates the contribution (e.g., claims of “bridging major gaps” or strong generalization). These should be softened to better reflect what is actually demonstrated.

Response:

We thank the reviewer for their observation. We agree that claims should be carefully aligned with the

empirical evidence and should not overstate the contribution.<br><br>In the revised manuscript, we have refined the language to ensure a more precise and balanced tone for our findings. Specifically, overly strong expressions such as “bridging major gaps” and “strong generalization” have been replaced with more accurate descriptions to reflect our findings.<br><br>For example, statements claiming that the model “bridges the gap between signal decomposition, graph representation learning, and reasoning capabilities” have been revised to more precise wording, such as “integrates signal decomposition, dynamic graph learning, and LLM-based modeling.” Similarly, claims of “strong generalization” have been replaced with expressions such as “stable performance across datasets.” These revisions have been applied throughout the manuscript to ensure that all claims are appropriately supported by the presented evidence.
Overall, the work has merit and is potentially publishable, but it requires revision to improve statistical rigor, clarify contributions, and refine presentation.

Reviewer #3:

1. Role and Necessity of the LLM

What specific capabilities of a pretrained Large Language Model are being exploited in this framework, given that the input consists of continuous traffic features rather than discrete linguistic tokens, and how does this differ from using a standard Transformer trained from scratch?

Response:

We thank the reviewer for this insightful question. Although the input consists of continuous traffic features, we use the pretrained LLM as a general sequence model rather than a language-specific model. The continuous inputs are projected into an embedding space compatible with the LLM, allowing it to process them as token-like representations and model both local and long-range dependencies through self-attention. The key advantage over a standard Transformer trained from scratch lies in the pretrained inductive biases and representation capacity of the LLM. Pretraining provides a strong initialization that enables more stable learning and better generalization, especially in small and noisy traffic datasets, where randomly initialized Transformers are more prone to overfitting.

We support this through comparative experiments with both pretrained (GPT-2, BERT, and DistilGPT-2) and non-pretrained backbones (GPT-2 and T5), where pretrained GPT-2 consistently achieves better performance. The results are shown below-

Table: *Comparative Analysis Across Different Pretrained and Non-Pretrained Backbones*

Backbone	MAE	MAPE	RMSE
T5 (not pretrained)	6.7799	47.17%	12.6522
GPT2 (not pretrained)	5.3352	38.61%	9.4408
BERT	5.3949	36.47%	9.8098
**GPT2 (our choice)**	**5.0742**	**36.17%**	**9.2021**
DistilGPT2	5.202	36.34%	9.5349

This indicates that the improvement comes from pretraining rather than the architecture alone.

2. Justification of Using GPT-2

Why is GPT-2 chosen as the backbone LLM for traffic forecasting, and how sensitive is the proposed framework to the choice of LLM architecture (e.g., GPT-2 vs. other Transformer variants)?

Response:

We thank the reviewer for their observation. GPT-2 was chosen as the backbone primarily due to its decoder-only, causal architecture, which aligns naturally with the traffic forecasting task, where future

values are predicted based solely on past observations. This allows the model to perform autoregressive sequence modeling without requiring additional architectural modifications. Additionally, GPT-2 has an optimal balance between pre-trained capacity and computational efficiency, making it suitable for our framework.

To assess the sensitivity of our framework to the choice of LLM backbone, we conducted comparative experiments with multiple Transformer variants, including BERT (encoder-only), T5 (encoder-decoder), DistilGPT-2, and a non-pretrained GPT-2. The results, presented in Figure 14 and discussed in the newly added subsection “Sensitivity to Backbone Choice” (Page 21), show that pretrained models consistently outperform non-pretrained ones, highlighting the importance of pretraining as below-

Sensitivity to Backbone Choice: Beyond numerical hyperparameters, the choice of the pre-trained backbone is critical for capturing temporal dependencies. We compare the performance of the pre-trained GPT-2 model, the pre-trained BERT model, the pre-trained DistilGPT-2 model, the GPT-2 model without pre-training, and the T5 encoder-decoder model without pre-training. As depicted in Fig. 11, the performance of the models with pre-training is comparatively better than that of the models without pre-training. Among all the models, the pre-trained GPT-2 achieves the best performance on the NYCTaxi dataset, with an MAE of 5.07 and an RMSE of 9.20. On the other hand, models trained without pretraining, including the randomly initialized GPT-2, exhibit substantially higher errors, indicating that architectural similarity alone is insufficient without pretrained knowledge. Although other pre-trained models, such as BERT and DistilGPT-2, achieve competitive results, the pre-trained GPT-2 model still performs slightly better. This is because BERT is an encoder-only model designed for bidirectional context, whereas the DistilGPT-2 model’s performance is limited by its capacity. This makes the pre-trained GPT-2 model an optimal choice for learning the temporal dependencies.

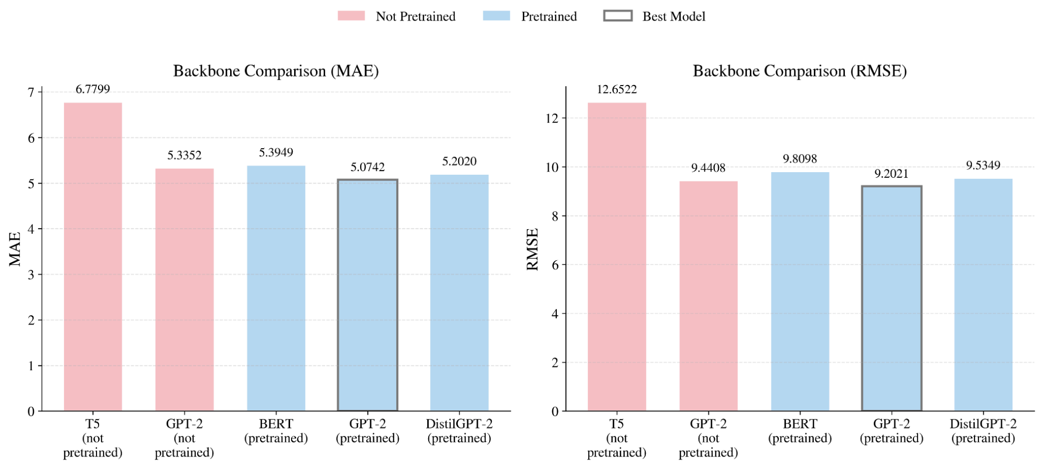

***Figure**: MAE and RMSE Comparison on Different Pretrained and Non-Pretrained Backbones*

Our results showed that it is possible to use other models as a backbone with competitive accuracy. However, GPT-2 consistently achieved the highest predictive accuracy.

3. Learning vs. Structural Bias

To what extent do the reported performance gains arise from the structural inductive biases introduced by VMD and dynamic graph masking, rather than from the pretrained knowledge embedded in the LLM itself?

Response:

We thank the reviewer for this important question regarding the relative contributions of structural inductive biases and pretrained representations. The performance gains in DG-LLM arise from the complementary roles of both components, and we have validated this through ablation and backbone sensitivity experiments.

The structural inductive biases (VMD and dynamic graph learning) improve performance by disentangling temporal patterns and capturing mode-specific spatial dependencies, thereby simplifying the learning problem. As shown in the ablation study (Page 20), removing these components leads to significant performance degradation, indicating that structural modeling plays a critical role.

On the other hand, the pretrained LLM contributes by providing strong sequence modeling capability and pretrained representations, which enable effective learning from the structured inputs. As shown in the backbone sensitivity analysis (Page 21), replacing the pretrained LLM with a non-pretrained Transformer results in noticeably worse performance, demonstrating the importance of pretraining.

These results indicate that the structural modules provide meaningful and disentangled representations, while the pretrained LLM enables effective modeling of these representations. The overall performance gains are therefore a result of their complementary interaction rather than being dominated by a single factor.

4. Interpretation of “Reasoning” in LLMs

The manuscript frequently refers to the “reasoning capabilities” of LLMs; can the authors clarify what form of reasoning is expected in this numerical forecasting setting and how it is empirically demonstrated?

Response:

We are thankful to the reviewer for pointing out this issue. We agree that the term “reasoning capabilities” can be misleading in the context of numerical spatiotemporal forecasting.

In our manuscript, this term was intended to describe the model’s ability to capture complex temporal dependencies and patterns based on historical observations through attention mechanisms, such as modeling long-range dependencies and distinguishing overlapping periodic behaviors. However, we acknowledge that this does not correspond to reasoning in the traditional semantic or logical sense associated with LLMs.

To avoid misinterpretation, we have revised the manuscript, replacing the word "reasoning capabilities" and other ambiguous terms with more precise descriptions, including “sequence modeling capability” and “long-range dependency modeling.” These changes have been applied throughout the manuscript to ensure clarity and accurate interpretation.

5. Dynamic Graph Stability and Identifiability

Since the dynamic adjacency matrix is learned directly from embeddings that themselves evolve during training, how stable and interpretable are the learned graphs across epochs and random seeds, and are multiple graph solutions equally valid?

Response:

We would like to express our gratitude to the reviewer for this thought-provoking question regarding the stability and identifiability of the learned dynamic graphs. We agree that the learned adjacency matrices are not strictly identifiable, as they are optimized to capture task-specific spatial dependencies rather than reconstructing the physical road network. Hence, it is possible that several graph configurations may perform equally well, and we did not impose the requirement that the learned structure must be unique.

As for the stability, we evaluated the model across five different random initialization seeds, and the structure of the learned graphs may differ across runs. However, the forecasting performance of the model remains highly consistent, as demonstrated by the tight 95% confidence intervals in Tables 3-5. The results are given below-

Table*: Multiseed Results with 95% interval for all Datasets*

Dataset	MAE Mean	MAE 95% CI	RMSE Mean	RMSE 95% CI	MAPE(%) Mean	MAPE(%) 95% CI
Taxi Drop	5.0594	[5.006, 5.113]	8.5906	[8.498, 8.684]	35.7	[35.28, 36.11]
Taxi Pick	5.2234	[5.180, 5.267]	8.7743	[8.693, 8.856]	34.76	[33.51, 36.00]
Bike Drop	1.6403	[1.630, 1.651]	2.3993	[2.373, 2.425]	45.49	[44.71, 46.26]
Bike Pick	1.7393	[1.726, 1.753]	2.6491	[2.615, 2.683]	48.17	[47.17, 49.17]
PEMSD04	18.8093	[18.19, 19.43]	28.2706	[27.78, 28.76]	13.84	[11.63, 16.05]
PEMSD08	14.5071	[14.20, 14.82]	22.6447	[22.19, 23.10]	10.96	[9.31, 12.60]

As for the interpretability, the learned graphs should be interpreted as functional connectivity graphs, as they represent task-relevant relationships rather than the physical network structure. These graphs capture the dynamic and mode-dependent interactions between nodes, which may differ from the physical connections but are beneficial for improving the performance of the forecasting model.

6. Risk of Information Leakage via VMD

Although VMD is applied offline per input window, can the authors formally justify that the decomposition process does not leak future information, especially given its optimization-based formulation?

Response:

We thank the reviewer for raising this important concern regarding potential information leakage. We confirm that the VMD decomposition does not introduce any future information leakage.

The dataset is first segmented into sliding windows, where each sample consists of an input sequence X of length T_{in} and a target sequence Y of length T_{out}. The VMD algorithm is then applied independently to each input window X only, without access to the corresponding future target sequence Y. Although VMD involves an optimization procedure, this optimization is performed locally within each input window and does not involve any future data points or global sequence information. As a result, the decomposition is strictly based on past observations and cannot leak information from the prediction horizon.

To clarify this explicitly, we have revised the Implementation Details section (Page 15) as follows-

VMD is applied to each window separately using only the input sequence X of length T_{in}, without including any future prediction window Y of length T_{out} to avoid any future information leakage.

7. Fairness of Baseline Comparisons

Given that DG-LLM leverages pretrained models with millions of parameters, while several baselines are trained from scratch, how do the authors ensure a fair comparison in terms of model capacity, pretraining advantage, and computational budget?

Response:

We thank the reviewer for pointing out this important aspect for fairness. Although DG-LLM utilizes a pretrained LLM backbone, we adopt a parameter-efficient fine-tuning strategy (LoRA), where more than 92% of the parameters remain frozen. As shown in Table 9, only approximately 7% of the total parameters are trainable (~18.4M), which keeps the effective training capacity comparable to that of many baseline models trained from scratch.

In order to further support the effectiveness of pretraining, we also compare DG-LLM with other LLM-based benchmark models, such as STLLM and STLLM+, that use a pretrained backbone. The results show that DG-LLM consistently achieves better performance than other pretrained LLM-based models (Table 3-4). This further supports that the improvement is not solely due to pretraining.

In addition, we have included a detailed analysis of computational cost in the newly added subsection "Computational Efficiency Analysis" (Page 22), where we report GFLOPs, parameter count, and inference latency across datasets. These results show that DG-LLM maintains moderate computational

complexity and achieves notable memory savings compared to full fine-tuning, ensuring a practical and competitive training budget.

***Table**: Computational efficiency of DG-LLM across datasets. Parameter counts are in millions (M).*

Dataset	Nodes	Total Params (M)	Trainable (M)	Train %	GFLOPs	Time/Epoch (s)	Latency (ms)
PEMSD04	307	257.81	18.47	7.17%	93.48	829.8	292
PEMSD08	170	257.7	18.37	7.13%	49.4	460	183
Bike Drop	250	257.76	18.43	7.15%	74.67	159.6	237
Bike Pick	250	257.76	18.43	7.15%	74.67	165	238
Taxi Drop	266	257.78	18.44	7.15%	79.89	176	253
Taxi Pick	266	257.78	18.44	7.15%	79.89	181	260

The subsection is added below-

DG-LLM adopts a parameter-efficient fine-tuning strategy, LoRA, in which only a small fraction of the model parameters are updated during training. Although the full model contains approximately 257M parameters, only about 7.15% (~18.4M) are trainable, while the majority of the pre-trained backbone remains frozen as shown in Table 8. This significantly reduces the effective optimization cost compared to full fine-tuning. In addition, Table 8 reports the computational efficiency across datasets, including GFLOPs, training time per epoch, and inference latency. Despite incorporating additional components such as VMD and dynamic graph learning, DG-LLM reduces RAM usage by 32.7% compared to full fine-tuning while maintaining moderate computational complexity and practical runtime performance. These results demonstrate that the proposed framework achieves a favorable balance between model capacity and efficiency for large-scale spatiotemporal forecasting.

8. Generalization Beyond Benchmarked Datasets

The evaluation is limited to six commonly used traffic datasets; how does the proposed method generalize to unseen cities, different sensor densities, or missing-sensor scenarios that are common in real-world deployments?

Response:

We thank the reviewer for pointing out this important aspect in real-world generalization. In our evaluation, we include six datasets with varying sensor densities and spatial configurations, ranging from sparse urban networks to dense highway sensor systems. In our revised version, we have

extended our evaluation process to include both robustness to missing data and generalization across datasets.

We have included a new subsection in our revised version dedicated to robustness to missing data (Page 22). In this section, we simulated missing values ranging from 10% to 50% and observed that our model maintains stable performance even under moderate levels of missing data. The subsection is added below-

To evaluate the robustness in handling incomplete observations, we conducted an MCAR (Missing Completely At Random) missingness simulation by randomly masking input sequences with missing rates of 10%, 20%, 30%, and 50%. The experiments were conducted across both Taxi Drop and Bike Drop scenarios and compared with our base model STLLM, where DG-LLM outperforms STLLM in all missing rates in MAE and RMSE metrics, as shown in Fig. 12.

The experiments demonstrated that DG-LLM performs steadily with increasing missing rates with gradual performance degradation, and maintains considerable accuracy in handling severe missing rates (50%). This further confirms that DG-LLM is more resilient to missing information and effectively exploits temporal and spatial information in handling incomplete data. In particular, for the Taxi Drop scenario, DG-LLM increases MAE from 5.0963 to 5.3216 with 10% missing information and from 5.4077 to 5.5624 with 50% missing information, while RMSE increases from 8.9431 to 9.6995 with 10% missing information and from 9.8142 to 10.4135 with 50% missing information. For Bike Drop, DG-LLM similarly increases MAE from 1.7422 to 1.9905 at 10% and from 1.8561 to 2.1172 at 50%; RMSE increases from 2.5974 to 3.0184 (10%) and from 2.8189 to 3.2852 (50%). In general, while both models perform worse with increasing missingness, DG-LLM maintains a stable margin over STLLM from moderate (20%) to severe (50%) missingness, which further justifies the robustness of our proposed design based on multi-scale temporal decomposition and dynamic graph learning.

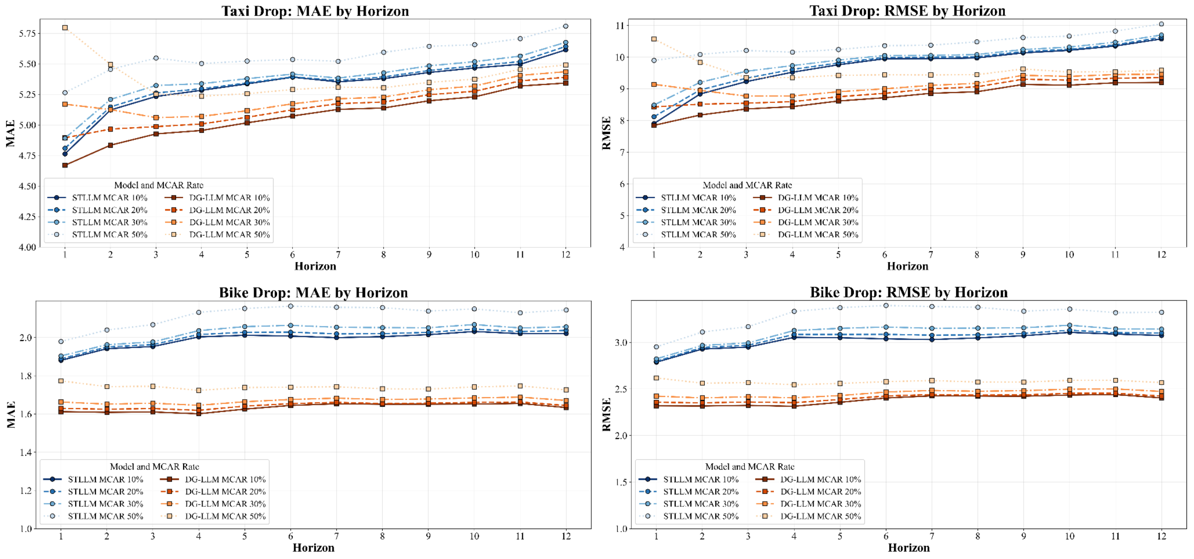

Figure: *MAE & RMSE Comparisons for Different Missing Rates on NYCTaxi and CHBike Datasets*

Moreover, we conduct a zero-shot generalization analysis across datasets with different sensor densities and urban structures. The results show that DG-LLM achieves reasonable transfer performance across similar domains (e.g., Bike Pick-up and Drop-off), while performance degrades when transferring between significantly different domains (e.g., urban taxi data to large-scale highway networks). This highlights both the adaptability of the model and the challenges of cross-domain generalization in heterogeneous traffic systems. This is added in the new “Zero-shot Generalization” subsection (Page 23) as added below-

Zero-Shot Generalization: To assess the robustness of the framework, we evaluated its performance through zero-shot transfer on different urban modalities and data scales. As shown in Fig. 16, the model demonstrates moderate transferability across similar domains and tasks, such as Bike Pick-up and Bike drop-off demand, with minimal degradation in MAE and RMSE performance. However, when we transfer from smaller-scale data sets such as NYCTaxi and CHBike to larger-scale and more complex PEMS highway network data, we see substantial degradation in performance. This is due to the difficulty in generalizing from low-density signals to high-density signals. The results show that the model is successfully able to learn the spatiotemporal patterns in the data, thus proving that graph adaptivity through VMD is possible even without training on the target network.

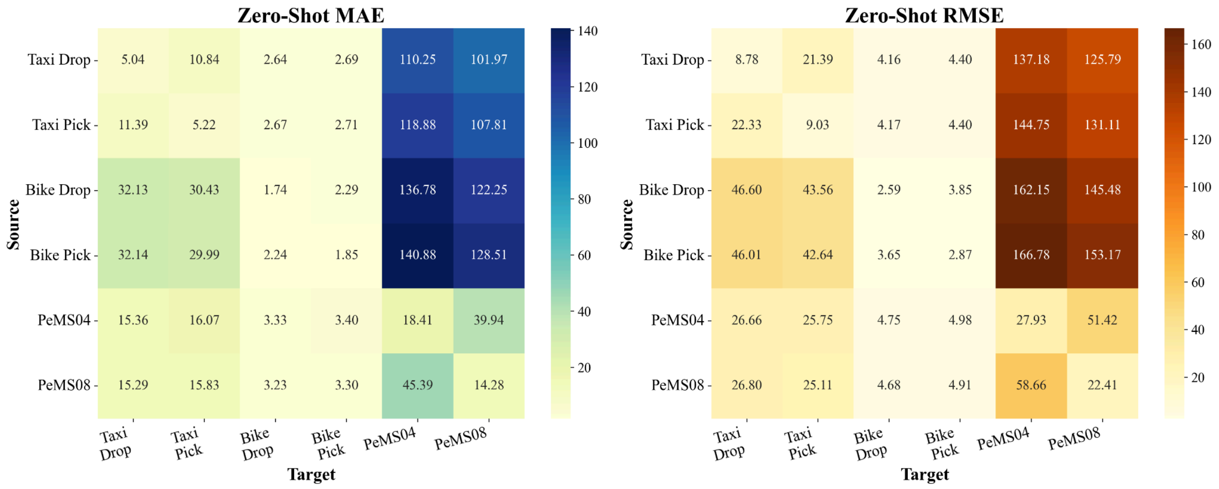

***Figure**: MAE and RMSE for zero-shot cross-dataset transfer performance across varying urban modalities and data scales*

We have added these analyses in new subsections on robustness and zero-shot generalization in the revised manuscript (Pages 22-23). While these results demonstrate the robustness and adaptability of DG-LLM under more realistic conditions, we acknowledge that generalization to entirely unseen cities without adaptation remains an open challenge, which we highlight as a direction for future work in the conclusion section (Page 24).

Reviewer #4:

This manuscript introduces a novel and sophisticated framework, DG-LLM, for spatiotemporal traffic forecasting, skillfully integrating Variational Mode Decomposition (VMD), dynamic graph learning, and a pretrained LLM backbone. The work is comprehensive and demonstrates strong empirical results. However, prior to publication, major revisions are required to clarify the model's novelty against very recent literature, justify specific design choices, and provide a more critical discussion of limitations.

1. The claimed novelty regarding the integration of VMD with LLMs for traffic forecasting needs clearer positioning against the immediate predecessor, STLLM+, and other recent works. A detailed ablation study or discussion is needed to isolate the contribution of the dynamic graph versus the decomposition. Is the performance gain primarily from VMD, the dynamic graph, or their synergy?

Response:

We thank the reviewer for pointing out the significance of properly differentiating our work from previous works. Recent LLM-based methods such as STLLM+ have shown promising results in spatiotemporal forecasting. However, these approaches typically operate on entangled time series signals and rely on static or globally shared spatial structures, which limit their ability to capture variations in spatial dependencies across different temporal patterns.

The novelty of our proposed DG-LLM does not lie in individual elements of the model, but rather in the joint consideration of frequency-dependent spatiotemporal structure. In particular, we propose to incorporate temporal decomposition with mode-specific dynamic graph learning, such that the model learns different spatial topologies for different frequency bands of the signal (e.g., trend, periodicity, and noise). The pretrained LLM then operates on these disentangled and structurally enriched representations, leading to more effective sequence modeling compared to directly processing raw, entangled signals. This allows us to incorporate frequency-dependent spatial heterogeneity, which is not addressed in previous LLM-based approaches.

We have revised the Introduction section (Page 3) to explicitly highlight this distinction and clarify the novelty of our approach. The added paragraph is provided below:

DG-LLM proposes a new paradigm for spatiotemporal forecasting by simultaneously disentangling frequency-specific temporal patterns and mode-specific spatial structures before employing the LLM for spatiotemporal prediction. Unlike previous approaches that employed LLMs on raw, entangled time series data or adopted a static graph, we leverage Variational Mode Decomposition (VMD) as a task-aware pre-processing component to disentangle the time series into intrinsic modes before the LLM models temporal patterns. As a result, the proposed approach can explicitly extract the intrinsic modes of the time series (i.e., trend, daily periodicity, and high-frequency noise). We further propose a mode-dependent dynamic spatial routing mechanism that learns separate graph topologies for each time series mode, as spatial correlations are not homogeneous across frequencies; long-range trends

and high-frequency noise have different dependency structures. By jointly modeling decomposed temporal patterns and their corresponding spatial structures, we capture multi-scale dependencies more effectively than existing approaches that rely on raw signals or static/global graph representations, thereby improving generalization across diverse datasets.

2. The choice of GPT-2 as the LLM backbone requires more justification. Given the focus on spatial-aware adaptation, were other architectures with inherent spatial bias (e.g., Vision Transformers adapted for graphs) considered? A brief discussion on the rationale for selecting a purely temporal, causal model would be helpful.

Response:

We thank the reviewer for this insightful comment regarding the architectural choice. While architectures such as Vision Transformers provide strong spatial inductive biases, they are typically based on bidirectional self-attention, which is not naturally aligned with the causal nature of time-series forecasting. In traffic prediction, the task requires forecasting future values based strictly on past observations, and bidirectional models must be carefully modified with masking to enforce temporal causality.

In contrast, GPT-2 is a decoder-only architecture with autoregressive masking, which inherently satisfies this causal constraint. This makes it a natural choice for modeling temporal dependencies without requiring architectural modifications.

In our model, spatial dependencies are incorporated via graph-constrained attention masks applied to token representations, and the pre-trained GPT-2 backbone provides strong sequential modeling capabilities. This provides us flexibility to inject spatial structure explicitly, rather than relying on implicit spatial bias within the backbone architecture.

3. The parameter analysis for VMD level K and unfrozen layers U is insightful. However, Figures 8 and 9 are referenced but not included in the provided text. These critical results must be present in the final manuscript to support the conclusions about optimal hyperparameters.

Response:

We apologize for this oversight. Figures 8 and 9 provide visualizations for “VMD level comparisons” and “Unfrozen layer comparisons”, respectively. These have been included in the Parameter Analysis Section (Page 21) of this revised version as Fig 9 and 10.

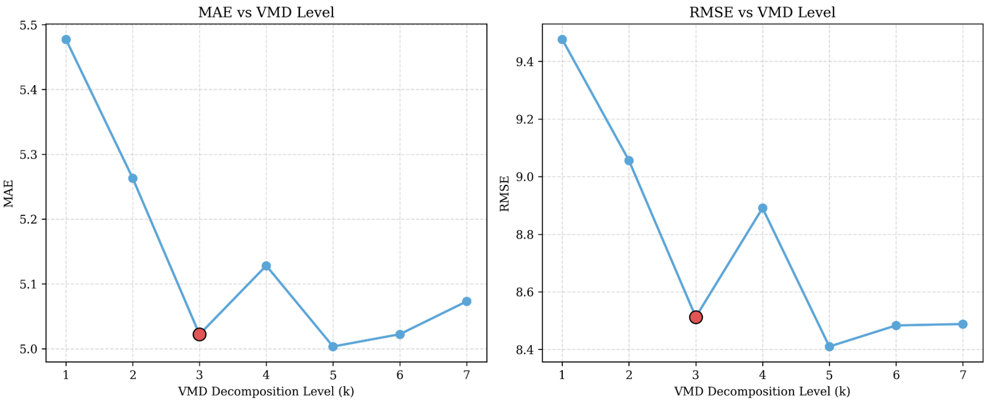

***Figure**: VMD level comparisons on the NYCTaxi Drop-off dataset*

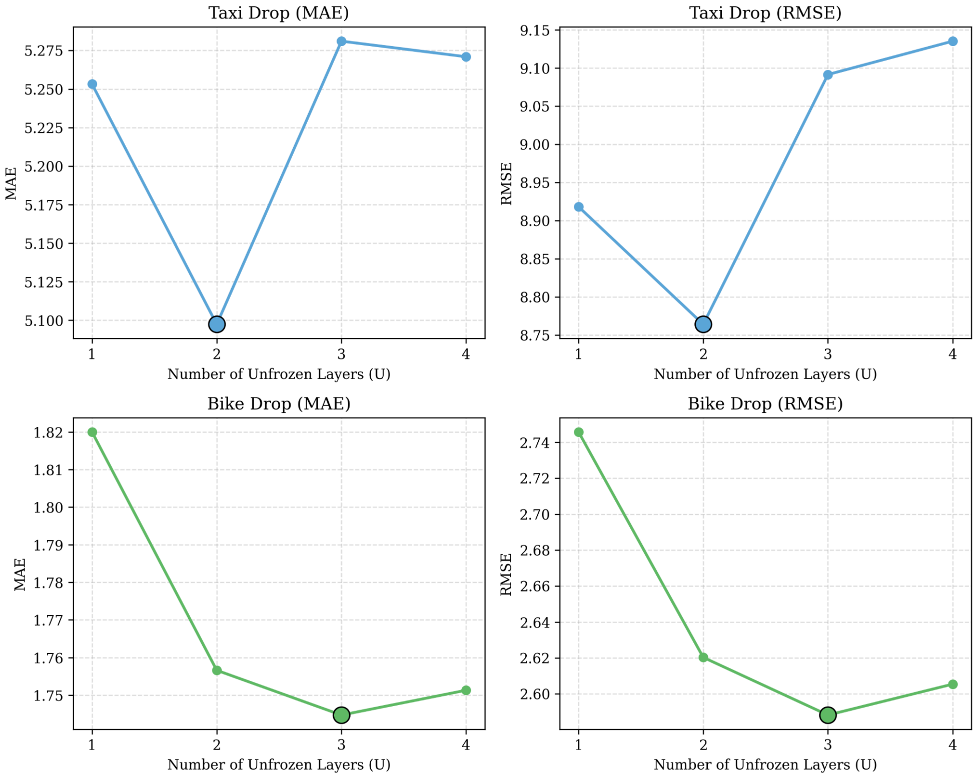

***Figure**: MAE & RMSE Comparisons for Different Unfrozen Layers on the NYCTaxi and CHBike Datasets*

4. The experiments use a fixed prediction horizon (T_out = 12) for short-term evaluation. The model's sensitivity to different horizon lengths should be analyzed. Does the advantage of VMD and the dynamic graph become more pronounced for longer-term forecasts (e.g., T_out > 12) compared to baselines?

Response:

We thank the reviewer for this important question regarding the sensitivity to the prediction horizon. In our experiments, DG-LLM is evaluated across multiple prediction horizons, ranging from

short-term(e.g., 30 minutes) to longer-term forecasts (e.g., 6 hours), as reported in the results tables. These results allow us to directly assess the model's performance as the forecasting horizon increases.

We observe that while most models perform comparably at very short horizons, the advantage of DG-LLM becomes more pronounced as the prediction horizon increases. This is because longer-term forecasting is more affected by the error accumulation due to entangled temporal patterns and evolving spatial dependencies. The proposed DG-LLM framework mitigates this through temporal decomposition (VMD), which separates frequency components, and dynamic graph learning, which adapts spatial dependencies across these modes. Together, these components enable more stable modeling of long-range spatiotemporal dependencies.

As shown in the long-horizon results in Table 6 (Page 19), DG-LLM maintains lower error compared to baseline models as the prediction horizon increases to T_{out}=96, demonstrating that the benefits of the proposed design are particularly significant in long-term forecasting scenarios.

5. While LoRA is used for efficiency, a concrete analysis of the parameter efficiency and training/inference speed compared to full fine-tuning of the LLM backbone is missing. Reporting the number of trainable parameters, training time per epoch, and inference latency would substantiate the practicality claims.

Response:

We thank the reviewer for this valuable suggestion. To address this, we have added a new subsection, "Computational Efficiency Analysis," in the revised manuscript (Page 22), where we provide a detailed analysis of parameter efficiency, training cost, and inference latency. All experiments were conducted on NVIDIA Tesla T4 GPUs (16GB memory). The parameter comparison table is given below-

Table*: Computational efficiency of DG-LLM across datasets. Parameter counts are in millions (M).*

Dataset	Nodes	Total Params (M)	Trainable (M)	Train %	GFLOPs	Time/ Epoch (s)	Latency (ms)
Taxi Drop	266	257.78	18.44	7.15%	79.89	176	253
Taxi Pick	266	257.78	18.44	7.15%	79.89	181	260
Bike Drop	250	257.76	18.43	7.15%	74.67	159.6	237
Bike Pick	250	257.76	18.43	7.15%	74.67	165	238
PEMSD04	307	257.81	18.47	7.17%	93.48	829.8	292
PEMSD08	170	257.7	18.37	7.13%	49.4	460	183

As shown in the table, although DG-LLM has approximately 257M total parameters, only about 7.15% (~18.4M) are trainable due to the use of LoRA, while the majority of the backbone remains frozen. In contrast, full fine-tuning would require updating all parameters, resulting in significantly higher optimization cost and memory usage. In addition to parameter efficiency, we report GFLOPs, training time per epoch, and inference latency across datasets. The results show that DG-LLM maintains practical efficiency, with training time ranging from approximately 160–830 seconds per epoch, depending on dataset size, and inference latency around 180–290 ms per sample. The subsection has been added below-

DG-LLM adopts a parameter-efficient fine-tuning strategy, LoRA, in which only a small fraction of the model parameters are updated during training. Although the full model contains approximately 257M parameters, only about 7.15% (~18.4M) are trainable, while the majority of the pre-trained backbone remains frozen as shown in Table 8. This significantly reduces the effective optimization cost compared to full fine-tuning. In addition, Table 8 reports the computational efficiency across datasets, including GFLOPs, training time per epoch, and inference latency. Despite incorporating additional components such as VMD and dynamic graph learning, DG-LLM reduces RAM usage by 32.7% compared to full fine-tuning while maintaining moderate computational complexity and practical runtime performance. These results demonstrate that the proposed framework achieves a favorable balance between model capacity and efficiency for large-scale spatiotemporal forecasting.

Reviewer #5:

This manuscript presents an ambitious and well-structured framework (DG-LLM) for spatiotemporal traffic forecasting by integrating signal decomposition, dynamic graph learning, and large language models. The work is technically sound, the experiments are comprehensive, and the results demonstrate competitive performance. However, to solidify its contribution and ensure clarity for the broader research community, the manuscript requires revisions to sharpen its novelty claim, provide deeper methodological justification, and engage more critically with the very recent wave of literature in this rapidly advancing field.

1. The novelty of integrating VMD with an LLM needs to be more precisely demarcated from contemporaneous works. The authors rightly position their work against STLLM+, but the field is evolving quickly. The introduction and related work sections should acknowledge and differentiate the core architectural philosophy of DG-LLM (dynamic graph injection) from other emerging paradigms for spatial-aware LLMs, such as those employing vision encoders or novel attention mechanisms, to clarify its unique value proposition.

Response:

We would like to thank the reviewer for pointing out the rapidly evolving landscape of spatially aware LLM architectures. In recent work, researchers have pursued different approaches to leverage spatial information in LLM-based forecasting models, including transforming traffic signals into images and feeding them into vision encoders, and using spatial attention mechanisms within the Transformer architecture.

However, the philosophy of designing DG-LLM is different from these works in that we focus on disentangling temporal patterns before spatial reasoning, rather than directly modifying the attention mechanism or using vision encoders. In particular, we utilize Variational Mode Decomposition (VMD) to decompose complex traffic signals into different frequency modes, each representing distinct temporal characteristics. We then use mode-specific dynamic graph learning for each frequency mode, enabling us to capture unique spatial patterns at different temporal scales. These are finally incorporated into the LLM using enriched token representations, which enable the LLM to capture spatiotemporal patterns while avoiding interference from mixed-frequency patterns in the original traffic signal. Thus, our work differs from existing spatial-aware LLMs that focus on modifying spatial attention within the Transformer architecture.

Following the reviewer's suggestion, we have expanded both the Introduction (Page 3) and Related Work sections (Page 4-6) to better position DG-LLM within the emerging literature on spatial-aware LLM architectures and to clarify its architectural distinctions.

2. The dynamic graph learning module is a cornerstone of the proposal. The description of the "curriculum-inspired training strategy" (blending static and learned graphs) is a practical solution but

lacks a theoretical or empirical justification. An ablation study quantifying the performance contribution of this blending strategy versus using only the learned dynamic graph would strengthen the argument for its necessity and illuminate its stabilizing effect during training.

Response:

We thank the reviewer for this constructive suggestion. The curriculum-inspired graph learning strategy is designed to improve training stability when incorporating learned dynamic topologies. In the early stages of training, the learned graph is initialized randomly and therefore provides unreliable spatial information. Relying solely on this evolving structure can introduce noise into the optimization process and lead to unstable training. To address this, the model initially leverages the static graph as a stable spatial prior and gradually increases reliance on the learned dynamic graph as it becomes more informative. This progressive transition stabilizes optimization while enabling the model to capture more complex non-Euclidean spatial relationships.

Following the reviewer's suggestion, we have added a new analysis (shown in Figure 8) comparing three configurations: (i) pure learned graph, (ii) pure static graph, and (iii) the proposed curriculum blending strategy. The result table is shown below-

***Table**: Result comparison of curriculum blending strategy*

Method	Dataset	MAE	MAPE	RMSE
pure learned	Taxi drop	5.0594	35.73%	8.7307
pure static		5.0396	36.65%	8.6414
**Curriculum (proposed)**		**5.0221**	**35.45%**	**8.5126**
pure learned	Bike drop	1.7703	47.67%	2.6656
pure static		1.7699	47.22%	2.6607
**Curriculum (proposed)**		**1.6474**	**45.21%**	**2.42**

The results show that the curriculum strategy consistently achieves the best performance across datasets (e.g., MAE 5.0221 on Taxi Drop and 1.6474 on Bike Drop), outperforming both pure static and pure learned variants. While the static graph provides stability but lacks flexibility, and the learned graph offers adaptability but suffers from instability during early training, the curriculum approach effectively combines the strengths of both. These findings empirically validate that the proposed blending strategy is not only necessary for stabilizing training but also contributes directly to improved forecasting accuracy.

We have included this analysis in the revised manuscript (Ablation Study Section, Page 20) to better justify the necessity of the proposed training strategy. The analysis is given below-

…However, directly learning a fully dynamic graph is unstable during training. As shown in Fig. 8, a curriculum learning strategy improves performance by facilitating a smooth transition from physical to learned spatial dependencies, integrating the static physical graph with the learned graph.

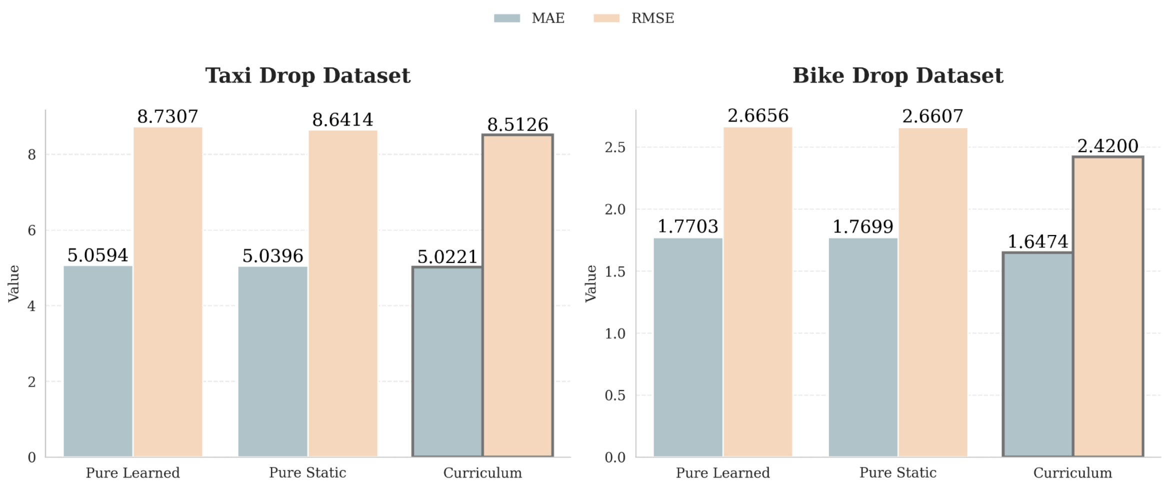

***Figure**: MAE and RMSE comparison of curriculum blending strategy on NYCTaxi and CHBike Dataset*

3. The paper demonstrates strong results but would benefit from a more rigorous analysis of its generalization capabilities. The authors should consult contemporary works that address the core challenges of adapting LLMs to spatio-temporal data and domain shifts. For a comprehensive understanding, I recommend reviewing Vision-LLMs for Spatiotemporal Traffic Forecasting, arXiv; Autoregressive data generation method based on wavelet packet transform and cascaded stochastic quantization for bearing fault diagnosis under unbalanced samples, Engineering Applications of Artificial Intelligence; Multi-domain weakly decoupled domain generalization network for fault diagnosis under unknown operating conditions, Knowledge-Based Systems. These works provide insights into alternative architectural paradigms (vision-language fusion), advanced signal processing for feature learning, and robust multi-domain generalization strategies. Engaging with these concepts will help contextualize DG-LLM's approach to handling spatial data and its robustness under distribution shifts, thereby strengthening the manuscript's methodological foundations.

Response:

We thank the reviewer for these insightful suggestions, as well as the mention of some relevant contemporary works on spatiotemporal modeling and domain generalization. In accordance with the reviewer’s suggestion, we have expanded the Related Work section (Pages 5-6) of the manuscript, including these approaches.

We compare vision-language fusion methodology in ST-Vision-LLM with our sample-wise temporal decomposition and graph-constrained attention. We also use the wavelet-packet-based CSQ-VAE and MWDG framework to further contextualize our use of VMD, as both methods demonstrate that frequency-domain decomposition is critical for learning features from complex sensor data. We will add the following citations in the revised manuscript in the Related Work section as follows-

“...Furthermore, contemporary approaches like Vision-LLMs [32] explore vision-language fusion to capture global spatial representations, suggesting that transforming spatiotemporal data into visual

modalities can enhance long-horizon forecasting and cross-domain generalization...”

“...Advanced signal processing techniques, such as wavelet packet transform combined with cascaded stochastic quantization [36], demonstrate that frequency-domain decomposition is critical for learning meaningful representations from complex sensor data. In addition, multi-domain weakly decoupled networks [37] highlight the importance of separating domain-specific and domain-invariant features for robust performance under distribution shifts…”

4. The efficiency claims related to using LoRA require substantiation. While LoRA is a known parameter-efficient fine-tuning method, the manuscript should include a concrete comparison. Reporting the total number of trainable parameters for DG-LLM versus full fine-tuning of the LLM backbone, along with comparative training times or FLOPs, would provide tangible evidence of the efficiency gains, which is crucial for assessing the framework's practicality.

Response:

We thank the reviewer for this important suggestion. To provide a concrete comparison, we have added a new subsection, “Computational Efficiency Analysis,” in the revised manuscript (Page 22), where we report detailed efficiency metrics in Table 8.

Table*: Computational efficiency of DG-LLM across datasets. Parameter counts are in millions (M).*

Dataset	Nodes	Total Params (M)	Trainable (M)	Train %	GFLOPs	Time/Epoch (s)	Latency (ms)
Taxi Drop	266	257.78	18.44	7.15%	79.89	176	253
Taxi Pick	266	257.78	18.44	7.15%	79.89	181	260
Bike Drop	250	257.76	18.43	7.15%	74.67	159.6	237
Bike Pick	250	257.76	18.43	7.15%	74.67	165	238
PEMSD04	307	257.81	18.47	7.17%	93.48	829.8	292
PEMSD08	170	257.7	18.37	7.13%	49.4	460	183

In a full fine-tuning setting, all parameters of the GPT-2 backbone (≈257M) would be updated during training. In contrast, DG-LLM updates only ≈18.4M parameters (about 7.15%) using LoRA, while more than 92% of the backbone remains frozen. This represents a substantial reduction in the number of trainable parameters compared to full fine-tuning. These results provide concrete evidence that the proposed framework achieves strong forecasting performance while maintaining high parameter efficiency. The subsection is given below-

DG-LLM adopts a parameter-efficient fine-tuning strategy, LoRA, in which only a small fraction of the model parameters are updated during training. Although the full model contains approximately

257M parameters, only about 7.15% (~18.4M) are trainable, while the majority of the pre-trained backbone remains frozen as shown in Table 8. This significantly reduces the effective optimization cost compared to full fine-tuning. In addition, Table 8 reports the computational efficiency across datasets, including GFLOPs, training time per epoch, and inference latency. Despite incorporating additional components such as VMD and dynamic graph learning, DG-LLM reduces RAM usage by 32.7% compared to full fine-tuning while maintaining moderate computational complexity and practical runtime performance. These results demonstrate that the proposed framework achieves a favorable balance between model capacity and efficiency for large-scale spatiotemporal forecasting.

5. The parameter analysis for the VMD level (K) and unfrozen layers (U) is valuable. However, the rationale for the selected ranges and the interpretation of the results could be more in-depth. For instance, why was K tested only up to 4? A discussion linking the optimal K=3 to the identifiable temporal patterns in traffic data (e.g., trend, daily periodicity, residual noise) would make the analysis more insightful.

Response:

We thank the reviewer for this suggestion. In the revised manuscript, we extended the parameter analysis and evaluated the VMD decomposition level up to K=7. The results show that performance improves as K increases from 1 to 3, indicating that moderate decomposition helps capture key temporal structures in traffic data. From a signal perspective, traffic time series typically consist of a small number of dominant components, including long-term trends, periodic patterns (e.g., daily cycles), and high-frequency fluctuations. A decomposition level of K=3 is sufficient to capture these primary temporal structures. Although slightly lower errors can sometimes occur for larger K values (e.g., K=5), the improvements are marginal, while the computational cost increases due to the additional decomposed signals. Therefore, K=3 provides a balanced trade-off between accuracy and efficiency, and we have clarified this discussion in the revised manuscript (Page 21). The results are added below-

***Table**: VMD level comparisons on the NYCTaxi Drop-off dataset*

K	MAE	RMSE	MAPE
1	5.4773	9.4766	38.03%
2	5.263	9.0559	37.91%
3	5.0221	8.5126	35.45%
4	5.1282	8.8909	35.88%
5	5.0034	8.4102	35.23%
6	5.0224	8.4834	35.02%
7	5.073	8.4883	35.95%

This result is illustrated using a graph (Figure 9) in the revised manuscript. The subsection is added below-

VMD Decomposition Level (K): For the VMD decomposition level K, we observe that increasing K from 1 to 3 on the NYCTaxi dataset improves our model's performance, as shown in Fig. 9. Specifically, the MAE and RMSE values are reduced to 5.02 and 8.51, respectively, from 5.48 and 9.48. This demonstrates that moderate decomposition helps the model capture meaningful temporal patterns in the traffic signals. However, further increasing K beyond 3 does not provide substantial performance benefits to our model. For example, K=5 produces a lower MAE of 5.00 than K=3, while increasing computational costs due to the larger number of decomposed signals. Therefore, we choose K=3 as a balanced configuration as it offers a high forecasting accuracy with lower computational overhead.

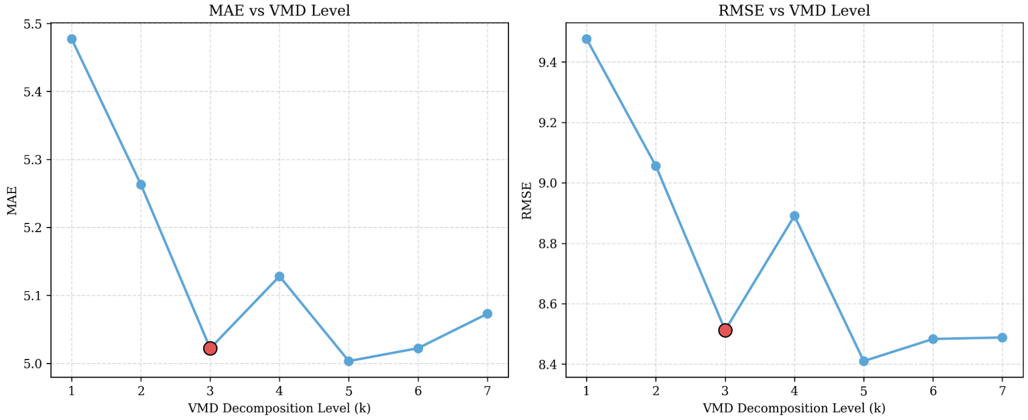

***Figure**: VMD level comparisons on the NYCTaxi Drop-off dataset*

6. The experimental design uses a fixed input and output horizon (e.g., T_in = T_out = 12). A sensitivity analysis demonstrating how the model's relative advantage over baselines changes with different prediction horizons (e.g., very short-term T_out=3 vs. long-term T_out=24) would be highly informative. It could reveal whether the benefits of decomposition and dynamic graphs are more pronounced for capturing long-range dependencies.

Response:

We thank the reviewer for this insightful suggestion. In our experimental setup, the model is trained under both short-term and long-term forecasting configurations, specifically T_{in}=T_{out}=12 and T_{in}=T_{out}=96. During evaluation, we report forecasting performance across multiple prediction horizons, as shown in Tables 3-6. This allows us to analyze model behavior from very short-term to longer-term forecasting scenarios.

From these results, we observe that while most models perform similarly at very short horizons, the prediction error of baseline methods increases more rapidly as the horizon grows. In contrast, DG-LLM maintains consistently lower MAE and RMSE across all horizons. We observe that training

with extremely short horizons (e.g., T_{out}=3) may lead to comparatively lower performance gains, as such settings do not fully capture longer-term temporal dependencies. In contrast, our experiments with longer horizons (e.g., T_{out}=96) demonstrate that DG-LLM is particularly effective when modeling extended temporal dynamics, where decomposition and dynamic graph learning provide the stronger benefit.

This indicates that the combination of temporal decomposition and dynamic graph learning helps the model better capture long-range temporal dependencies, which becomes particularly beneficial for longer forecasting horizons.
