# Supplementary material for: DG-LLM: Decomposition-based dynamic graph adaptation of large language models for spatiotemporal traffic forecasting
Source: PLoS One. 2026 May 19;21(5):e0349527. doi: 10.1371/journal.pone.0349527 (PMC13186391; doi:10.1371/journal.pone.0349527)
Supplement: Attachment — Submitted filename: Response to reviewers v2.pdf [file pone.0349527.s004.pdf]

Response to Reviewers' Comments

**Journal:**	PLOS ONE
**Manuscript ID:**	PONE-D-26-00024R1
**Title of Paper:**	DG-LLM: Decomposition-based dynamic graph adaptation of large language models for spatiotemporal traffic forecasting
**Authors:**	Sadia Tabassum, Naushin Nower
**Date Sent:**	April 15, 2026

We sincerely thank the reviewer for the thorough and constructive evaluation of our manuscript. The insightful comments and suggestions have been invaluable in refining the clarity, quality, and overall presentation of our work. In response, we have addressed all the points and made corresponding revisions throughout the manuscript. For ease of reference, all modifications have been highlighted in blue in the revised manuscript. We greatly appreciate having an opportunity to improve the quality of our manuscript through your constructive suggestions.

Reviewer #6:

This study proposes DG-LLM, a spatiotemporal traffic forecasting framework that combines signal decomposition, dynamic graph learning, and pretrained large language models with efficient fine-tuning to capture multi-scale temporal patterns and spatial dependencies across diverse traffic datasets. It requires a major revision based on:

1. The abstract is overly dense with methodological components (e.g., decomposition, dynamic graphs, LLM integration, LoRA), which obscures the core novelty; the authors should streamline the description and clearly emphasize the primary contribution and innovation.

Response:

Thank you for this valuable observation. We have revised the abstract to improve readability by simplifying the description and focusing more clearly on the primary contribution and innovation of the proposed DG-LLM framework **(Page 1)**. The revised part of the abstract paragraph is provided below:

… In this paper, we present a novel framework named DG-LLM that leverages the advantages of decomposed temporal representations and adaptive spatial connectivity to model spatiotemporal dependencies. In this framework, traffic signals are decomposed into intrinsic modes, and dynamic graphs are learned for each mode to represent the spatial dependencies. These representations are then incorporated with pre-trained Large Language Models for effective long-range temporal dependency modeling.

2. Although relative improvements in MAE and RMSE are reported, the abstract lacks baseline references, dataset scale, and evaluation protocol details; providing this context is necessary to properly assess the significance and robustness of the reported gains

Response:

We appreciate this suggestion. In the revised abstract, we have incorporated additional contextual information, including references to baseline models, the datasets used, and a brief indication of the evaluation setting **(Page 1)**. This provides a clearer understanding of the significance and robustness of the reported improvements. The revised part of the abstract paragraph is provided below:

… We conducted comprehensive experiments across six real-world traffic datasets spanning urban mobility systems and highway traffic networks and evaluated short- and long-term forecasting. Experimental results demonstrate that our framework provides significant improvements over state-of-the-art approaches, including benchmark graph- and LLM-based spatiotemporal forecasting models, even in long-term forecasting scenarios with severe temporal instability. Our model outperforms other methods by achieving 13-19% improvements in MAE and 19-25% in RMSE across all six benchmarks compared with baseline approaches. Additional analyses, including ablation

studies, robustness to missing data, and zero-shot cross-dataset evaluation, further validate the effectiveness and generalization capability of the proposed framework.

3. The introduction should clearly conclude with a distinct section highlighting the novel contributions of your work.

Response:

Thank you for the comment. We have revised the final part of the Introduction to more clearly and explicitly present the novel contributions of this work **(Pages 3-4)**. The contributions are now described in a concise and structured manner to improve clarity. The revised paragraph is provided below:

The major contributions of this work are summarized as follows:

- In this paper, we propose a decomposition-based spatiotemporal forecasting framework that models multi-scale temporal patterns and their corresponding spatial dependencies, thereby addressing the limitations of existing methods that operate on entangled time series.
- Within this framework, we design a mode-dependent dynamic graph learning mechanism that constructs a separate graph for each decomposed temporal mode to capture the homogeneous patterns, enabling adaptive modeling of traffic interactions across different frequency components.
- We further integrate these mode-specific graphs into a graph-aware pretrained large language model, enabling it to leverage dynamic spatial structures while effectively capturing long-range temporal dependencies.
- We validate the effectiveness of the proposed framework through a comprehensive experimental evaluation across various benchmark real-world datasets, showing significant improvements in both short- and long-term forecasting scenarios.

4. At the ending of the intro, it is advised to add a para that mentions briefly what each next section contains.

Response:

We agree that this improves readability. A new paragraph has been added at the end of the Introduction **(Page 4)**, briefly describing the organization of the manuscript and the contents of each section. The added paragraph is provided below:

The remainder of this paper is organized as follows. The Related Work section discusses previous works on graph-based, LLM-based, and decomposition-based approaches. The Problem Statement section introduces problem formulation and notations. The Methodology section presents the proposed DG-LLM framework. In the Experiments Section, we discuss our dataset, baselines, evaluation metrics, and experimental setup. The Results section presents the experimental results for both short-term and long-term forecasting, along with ablation studies and additional analyses. The

Discussion section presents the findings, and the Conclusion section concludes the paper, outlining future research directions.

5. The literature review should benefit from more explorations of previous studies.

Response:

We appreciate the reviewer's valuable feedback. The Related Work section has been substantially revised and expanded to provide a more comprehensive exploration of prior studies **(Pages 4-6)**.

The revised section now includes a structured discussion of the evolution of traffic forecasting methods, ranging from classical statistical models (e.g., ARIMA, Kalman filtering) to modern deep learning paradigms, including graph-, LLM-, and decomposition-based architectures, highlighting their respective strengths and limitations. We have also incorporated additional relevant works on traffic forecasting and LLM-based approaches to broaden the literature review.

We further refined the analysis within each category by emphasizing key methodological developments and existing limitations. The research gap has also been more clearly articulated by contrasting graph-based, LLM-based, and decomposition-based approaches, emphasizing their limitations in jointly modeling multi-scale traffic patterns and dynamic spatial dependencies.

6. The discussion section needs to be expanded to more thoroughly analyze the results.

Response:

We sincerely appreciate the reviewer's valuable comment. In our revised paper, the Discussion section has been significantly improved, with further analysis of the experimental findings **(Pages 23-24)**. We added a deeper interpretation of the learned mode-dependent graph structures, explained the complementary contribution of the components to the observed forecasting gains, and discussed dataset-specific behavior across urban demand and highway traffic settings. We also strengthened the analysis of long-horizon forecasting stability and zero-shot generalization, and explicitly highlighted the limitations and transferability challenges of the proposed framework. The revised Discussion section is provided below.

To gain deeper insights into the proposed framework, we analyze its learned spatiotemporal representations, forecasting behavior, and generalization characteristics.

Spatial Graph Variations: The learned adjacency matrices corresponding to the three temporal modes using the VMD algorithm are shown in Fig. 13. It is evident from the figure that the relationship between the nodes depends on the frequency associated with the data. The nodes are strongly connected for low-frequency modes representing long-term and trend-based information. The patterns are caused by the general behavior of traffic dynamics in the whole network, such as daily commuting. In contrast, high-frequency modes representing sudden events, like accidents or bad weather, exhibit a sparse relationship between the nodes. The sudden changes impact a limited area of

the network. Therefore, the nodes experience congestion only in nearby nodes. Thus, there exist variations in the dependencies of nodes on different temporal scales. Decomposing the data to isolate homogeneous components enables the model to learn distinct spatial interactions for each mode independently.

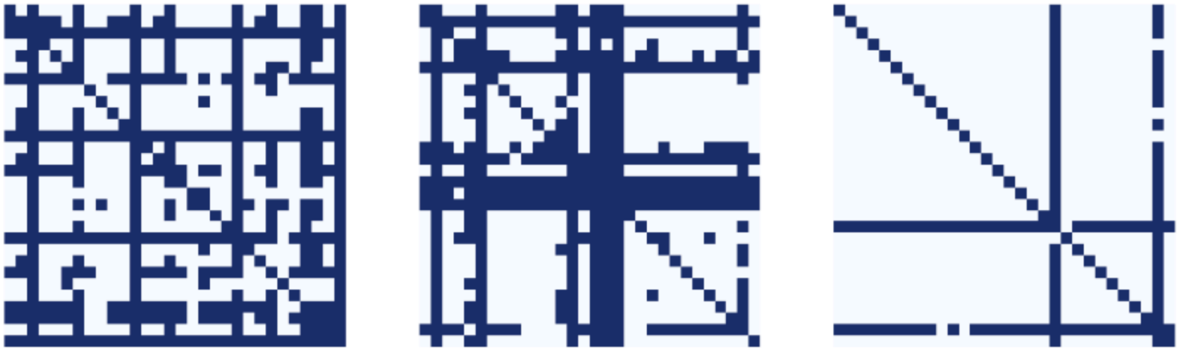

(a) **(b)** **(c)**

Fig 13: Mode-dependent graphs learned from decomposed traffic signals. (a) Low Frequency, (b) Mid Frequency, and (c) High Frequency

Forecasting Stability: Fig. 14 shows the trajectories of short- and long-term predictions at 12- and 96-horizon forecasting horizons, respectively. While baseline methods often fail to capture sharp fluctuations during peak intervals, our model remains closely aligned with the ground truth. The stability achieved at longer horizons (e.g., 48 hours) indicates a significant reduction in cumulative error. This suggests that temporal decomposition helps preserve long-term structural patterns, while graph-aware attention ensures the model remains responsive to local temporal variations, leading to more stable long-horizon predictions.

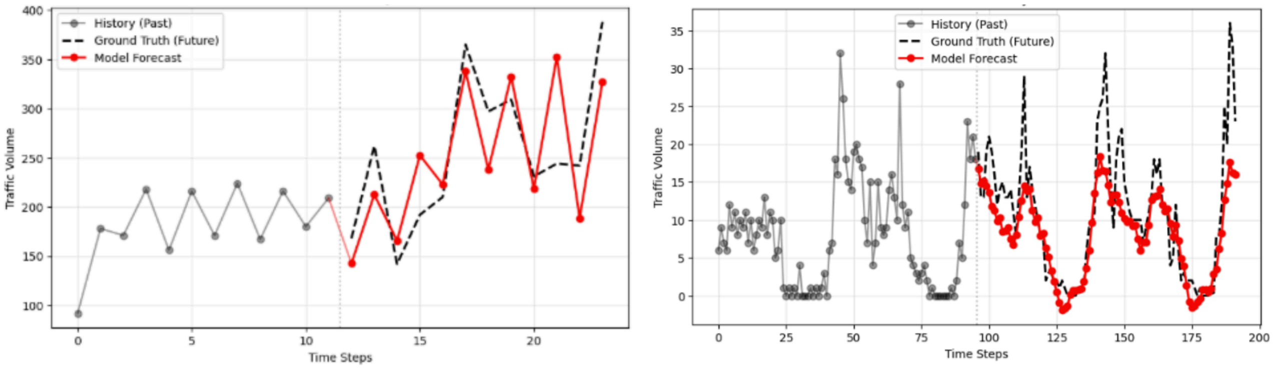

(a) **(b)**

Fig 14: Traffic forecasting visualization: (a) Short-term, (b) Long-term.

To further demonstrate the effectiveness of the proposed model in capturing periodic patterns, we present continuous predictions across the entire test dataset for three benchmark datasets (Taxi drop-off dataset, CH-Bike drop-off dataset, and PeMS04 dataset) as shown in Fig 15. The results show that the proposed model maintains high accuracy consistently across all datasets over the full test

horizon, including periodic trends such as morning and evening rush hours. This indicates that the model preserves long-term temporal structures while accurately modeling daily and weekly patterns, enabled by disentangling temporal components and reducing interference across different time scales.

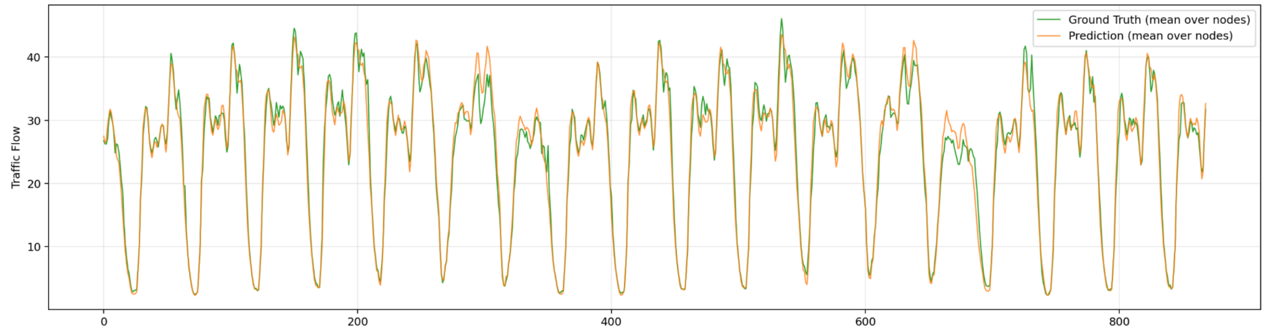

(a)

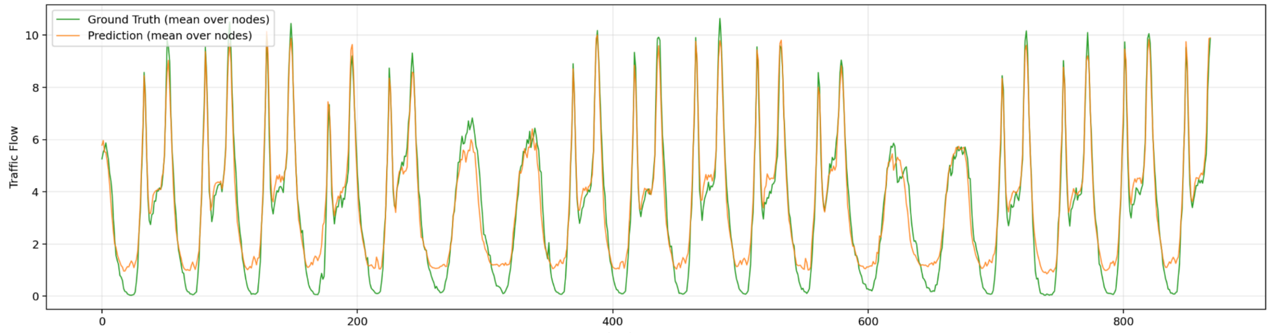

(b)

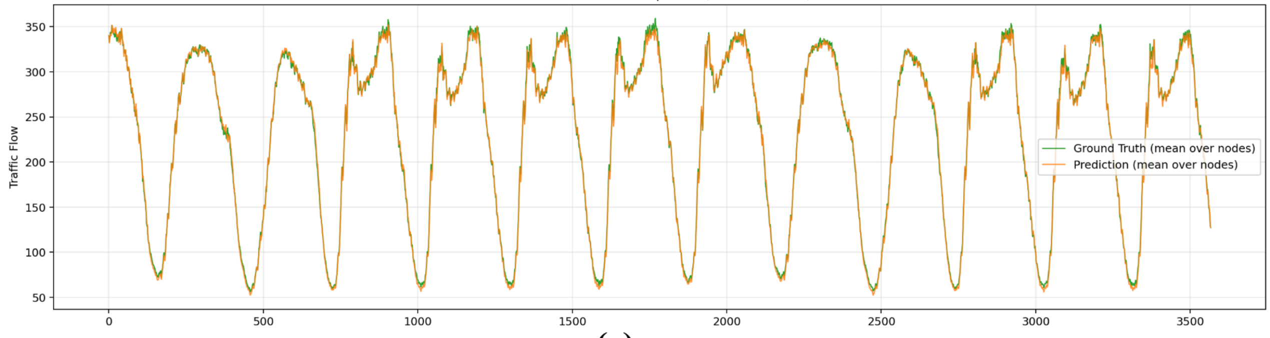

(c)

Fig 15: Traffic forecasting results: (a) Taxi drop-off dataset, (b) CH-Bike drop-off dataset, (c) PeMS04 dataset.

Zero-Shot Generalization: To assess robustness, we evaluate zero-shot transfer by training the model on one dataset and testing it on the remaining datasets without any fine-tuning. This process is repeated across all six datasets for cross-dataset evaluation. As shown in Fig. 16, the model demonstrates moderate transferability across similar domains and tasks (e.g., Bike Pick-up to Bike Drop-off demand), with minimal degradation in MAE and RMSE performance. However, when we transfer from smaller-scale datasets (e.g., NYC-Taxi and CH-Bike) to larger-scale or more complex datasets (e.g., PEMS highway data), we observe substantial performance degradation. This is due to

the difficulty in generalizing from sparse data to dense data. This indicates that while the model learns transferable temporal patterns, its performance remains sensitive to differences in topology and data distribution. This suggests that the learned representations remain partially dependent on the spatial characteristics of the training data.

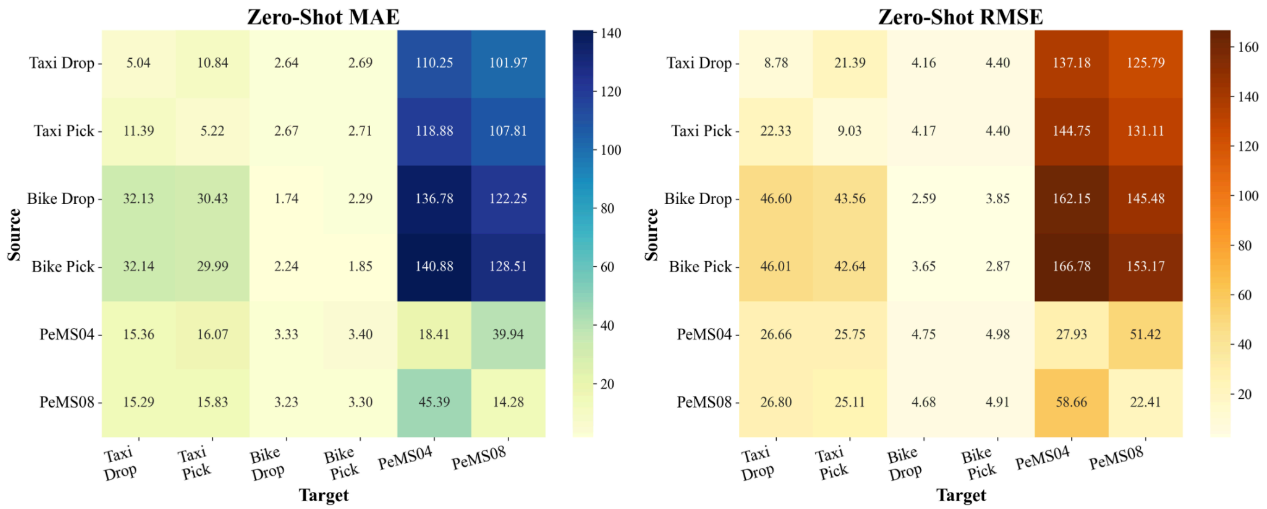

Fig 16: MAE and RMSE for zero-shot cross-dataset transfer performance across varying urban modalities and data scales.

These findings highlight that traffic dynamics are inherently multiscale, with spatial dependencies varying across frequency components. Therefore, modeling these variations is critical for robust long-horizon forecasting.

7. The first paragraph of the conclusion should succinctly summarize the contributions of the study in past tense.

Response:

Thank you for the suggestion. The first paragraph of the Conclusion has been revised to provide a concise summary of the study in the past tense, clearly reflecting the study's contributions and outcomes **(Page 25)**. The revised paragraph is provided below:

In this study, we proposed DG-LLM, a novel spatiotemporal forecasting architecture that employed decomposition-based temporal modeling and adaptive spatial relationships to represent traffic dynamics. This method disentangled traffic signals into intrinsic temporal components and learned corresponding dynamic graph representations that helped model multi-scale spatiotemporal patterns and dynamic dependencies in traffic signals. It addressed the limitations of existing methods that rely on entangled traffic signals or static spatial relationships in traffic data representations. Experimental results demonstrated that the proposed framework achieved consistent improvements over state-of-the-art methods, particularly in long-horizon forecasting scenarios where temporal instability is more pronounced. The results highlighted the importance of adaptive spatial connectivity in the

representation of traffic data. Incorporating dynamic graph learning proved effective for modeling irregular road networks and heterogeneous mobility patterns. Constraining the LLM's attention mechanism through graph-aware attention masks guided the model toward spatially relevant interactions. In addition, a pretrained LLM backbone was efficiently leveraged via LoRA, reducing the fine-tuning parameter cost.

8. The second paragraph of the conclusion should provide clear and actionable future recommendations.

Response:

We have revised the second paragraph of the Conclusion to include clear, actionable directions for future research **(Page 25)**. These revisions provide a more forward-looking perspective on potential extensions of this work. The revised paragraph is provided below:

Despite its effectiveness, the proposed model still has limitations when transferred across structurally distinct traffic networks, resulting in decreased cross-domain generalization performance. This highlights the challenge of variations in topology, sensor density, and mobility patterns, which can be difficult to generalize across. Also, while parameter-efficient fine-tuning reduces training overhead, computational efficiency remains a concern due to the sequential nature of decomposition and the complexity of attention mechanisms. Future research would involve developing strategies for cross-domain generalization and transfer learning, in addition to enhancing scalability via sparse attention mechanisms and optimized input embedding for real-time deployment in large-scale urban systems.

9. Equations are not properly cited, please add original references.

Response:

We appreciate this valuable comment. In the revised manuscript, we have carefully reviewed all equations and added appropriate citations to their original sources (e.g., VMD, attention mechanism, graph attention, and LoRA). In addition, we have a dedicated appendix (S1 Appendix: Preliminaries) that provides a more detailed formulation and discussion of these foundational methods with proper attribution **(Pages 26-28)**.

10. Please include the response letter separately with different colors.

Response:

We thank the reviewer for this suggestion. The response letter has been prepared separately in accordance with *PLOS One* guidelines, with distinct color formatting used to clearly differentiate reviewer comments and author responses.
